# Updated concepts of seismic gaps and asperities to assess great earthquake hazard along South America

**DOI:** 10.1073/pnas.2216843119

**Published:** 2022-12-13

**Authors:** Thorne Lay, Stuart P. Nishenko

**Affiliations:** ^a^Department of Earth and Planetary Sciences, University of California Santa Cruz, Santa Cruz, CA 95064

**Keywords:** asperities, seismic gaps, slip deficit, South American large earthquakes, seismic hazards

## Abstract

So far in this century, six very large–magnitude earthquakes (*M_W_* ≥ 7.8) have ruptured separate portions of the subduction zone plate boundary of western South America along Ecuador, Peru, and Chile. Each source region had last experienced a very large earthquake from 74 to 261 y earlier. This history led to their designation in advance as seismic gaps with potential to host future large earthquakes. Deployments of geodetic and seismic monitoring instruments in several of the seismic gaps enhanced resolution of the subsequent faulting processes, revealing preevent patterns of geodetic slip deficit accumulation and heterogeneous coseismic slip on the megathrust fault. Localized regions of large slip, or asperities, appear to have influenced variability in how each source region ruptured relative to prior events, as repeated ruptures have had similar, but not identical slip distributions. We consider updated perspectives of seismic gaps, asperities, and geodetic locking to assess current very large earthquake hazard along the South American subduction zone, noting regions of particular concern in northern Ecuador and Colombia (1958/1906 rupture zone), southeastern Peru (southeasternmost 1868 rupture zone), north Chile (1877 rupture zone), and north-central Chile (1922 rupture zone) that have large geodetic slip deficit measurements and long intervals (from 64 to 154 y) since prior large events have struck those regions. Expanded geophysical measurements onshore and offshore in these seismic gaps may provide critical information about the strain cycle and fault stress buildup late in the seismic cycle in advance of the future great earthquakes that will eventually strike each region.

## Significance

Earthquakes involve complex, nonlinear frictional instabilities and dynamical processes that undermine deterministic predictability. Nonetheless, plate boundary strain energy budgets, driven by long-term relative plate motions, provide a degree of cyclicity in occurrence of very large earthquake ruptures on subduction zone plate boundary (megathrust) faults. For the largest earthquakes, a basic cycle of interseismic fault locking and strain accumulation, abrupt coseismic fault sliding and strain energy release, and postseismic stress adjustment occurs, basically compatible with the elastic-rebound theory of faulting. Heterogeneous slip and triggering interactions give rise to irregularity in this seismic cycle, but by quantitatively characterizing the slip in very large earthquakes in regions that have previously ruptured in large historic earthquakes, improved understanding of future earthquake hazards is possible.

Earth’s largest earthquakes occur on subduction zone plate boundary faults, or megathrusts, where stick-slip sliding accommodates convergent relative plate motions. Long-term relative plate motions result in episodic stress buildup and elastic strain accumulation on either side of frictionally locked portions of the megathrusts followed by abrupt fault sliding offsets and surrounding strain energy release in large earthquakes as the system strives to keep up with the long-term relative plate motions. The underlying conceptual framework dates back to the elastic-rebound theory that emerged from the 1910 work of Reid (1) following the 1906 San Francisco earthquake and the recognition of large-scale plate tectonics in the 1960s. Uncertainties in stress drop relative to absolute stress levels, variability in failure stress level, fluctuations in fluid pressure distributions, nonlinear frictional instabilities, complexity of megathrust physical properties, and adjacent earthquake stress interactions (2) result in space and time irregularities of very large megathrust earthquake occurrence. Nonetheless, as earthquake observations continue to accumulate, there has been substantial progress in understanding megathrust earthquake hazard in the context of the tectonic strain energy budget for the system; the so-called Reid renewal interval of strain reaccumulation that must occur before another very large earthquake ruptures a given portion of the plate boundary.

**Fig. 1.**
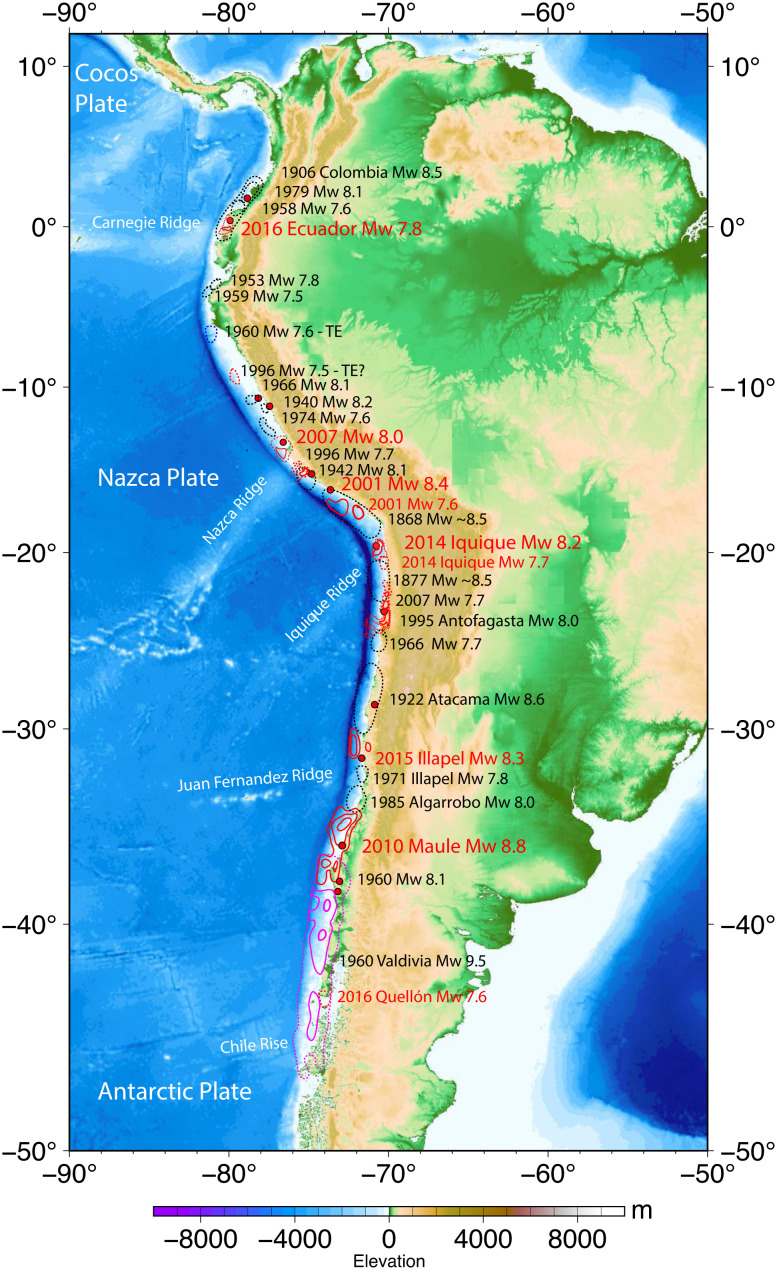
The most recent large earthquake rupture zones (*M_W_* ≥ 7.5) along each region of the west coast of South America where the Nazca plate is underthrusting the continent. Black dashed regions indicate aftershock zones for older events (black labels); red contours indicate slip distribution for large events in this century (red labels) from references in the supplement; purple contours indicated slip contours (1, 10, and 20 m) for the 1960 Valdivia, Chile, event.

The focus here is on the subduction zone extending ~6,500 km along the western coast of South America, where the Nazca plate is underthrusting the South American plate. The occurrence of 6 very large megathrust earthquakes (*M_W_* > 7.8) along this plate boundary during the last 21 y (Fig. 1) has reinforced several fundamental observations that were made about great earthquake occurrence more than 50 y ago:

• The rupture zones of major earthquakes along geometrically simple megathrusts tend to abut without significant overlap.
• Very large earthquakes (*M_W_* ≥ 7.8) have a tendency to occur along portions of the megathrust where comparable size earthquakes have not occurred for many decades or even several centuries (3, 4). These regions are called seismic gaps.

**Fig. 2.**
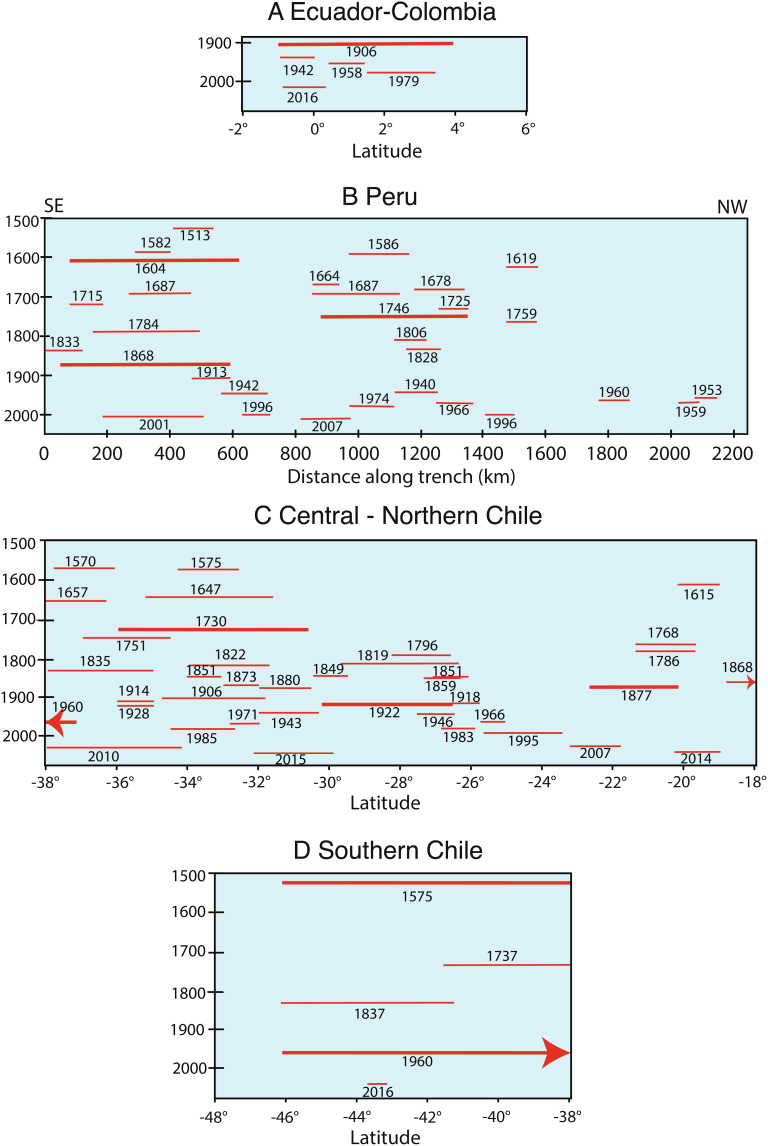
Along–plate boundary rupture distributions for historic large earthquakes (*M* ≥ ~7.5) in (*A*) Ecuador–Colombia, (*B*) Peru (6), (*C*) central to northern Chile (7–9), and (*D*) southern Chile (7, 8). Bolder lines represent Breakthrough Ruptures that likely span the entire width of the plate boundary.

The first point is readily evident in the nearly continuous distribution of the most recent large earthquake rupture zones along the entire boundary depicted in Fig. 1. The large events since 2000 are shown with coseismic slip contours that emphasize the nonuniform slip along dip and along strike of the subduction zone. Most of the Nazca–South America plate boundary has produced repeated large earthquakes along the full distribution of ruptures shown in Fig. 1. The second point above is demonstrated by considering the estimated along-strike extent of large historic earthquakes (*M_W_* ≥ ~7.5) along the South American subduction zone shown in Fig. 2. It is important to note that there are examples where recent very large earthquakes have ruptured smaller areas than in prior events (this is notable for the 2016 Ecuador earthquake (Fig. 2*A*), which reruptured the 1942 zone but only ruptured the southern portion of the 1906 zone, and the 2001 southern Peru event (Fig. 2*B*), which ruptured about 2/3 of the length of the 1868 event, as well as examples where earthquakes have ruptured areas larger than in prior events (the 2010 Maule, Chile, earthquake (Fig. 2*C*) ruptured the 1928 zone plus most of the 1835 zone). One has to be cautious about inferring overlap of two-dimensional ruptures, as for the case of the 2007 northern Chile rupture, located on the down-dip portion of the megathrust, which may not overlap shallow rupture in the 1877 event (5). Nonetheless, it is clear that absolute segmentation does not exist, and ruptures can comprise multiple adjacent portions of the boundary or not, an aspect that was not well recognized in the early seismic gap discussions.

About 43 y ago, an additional key concept involving slip heterogeneity in megathrust earthquakes developed from observations of variations in maximum earthquake size and complexity of seismic waves radiated from very large earthquakes in different subduction zones. Regions on the fault with large coseismic slip and associated large volumetric strain release are identified as “asperities,” borrowing a contact mechanics term for the point contacts of microscale surface interactions (10–12). Patchy distributions of large-slip regions during large earthquakes have been affirmed by increasingly well-resolved finite-fault slip models, but whether the underlying cause is material property variations (sediments/rock contacts), boundary roughness (seamounts/horst and graben structures), or hydrologic variations (pore fluids), or some combination of these factors, and their persistence over multiple events is still an active area of research. A somewhat complementary perspective of earthquake ruptures being controlled by portions of the fault that delimit sliding, or “barriers,” was also advanced about this time (13). The connection between asperities, barriers, and gaps is intrinsically complex as heterogeneity of stress and strain accumulation and variable frictional properties complicate the notion of a fault “sticking,” which is intrinsic to the elastic-rebound theory (14). While some faults may actually lock up uniformly over their entire seismogenic surface and rupture accordingly, others may have patchy locking and irregular failure with mixed seismic and aseismic modes of boundary sliding, leading to distributions of event size on the same megathrust, partial rupture within a seismic gap, and variability in great earthquake size in a given region.

While the early conceptual models of seismic gaps and asperities have guided many analyses of large earthquakes over the past decades, major advances over the past 30 y in understanding the complexity of frictional behavior, development of geodetic methods for directly detecting interseismic strain accumulation in the upper plate of subduction zones, and joint seismic–geodetic–tsunami analyses of finite-fault slip distributions have provided a more physical context for understanding heterogeneity of slip on faults. The inferred “patchiness” of megathrust geodetic locking and large event slip irregularity give a better understanding of why large event ruptures tend not to overlap with recent events and why some events can rupture regions that at other times fail in several discrete events. Stress shadowing along dip and along strike can result in slip deficit before and after large events in regions that are not mechanically coupled (15).

We draw on the updated perspectives of seismic gaps, persistent asperities, and geodetic locking to evaluate the current state of seismic hazard for very large earthquakes along the Nazca–South American megathrust. Our focus is on very large event hazards (*M_W_* ≥ 7.8). These very large events release the majority of accumulated tectonic strain over large enough portions of the plate boundary (~120 km × 40 km) for Reid renewal models to be applicable. Smaller ruptures can have adjacent rupture patches that may not involve rerupture of a common megathrust region making them more ambiguous to interpret. The identification of seismic gaps for very large events along the South American subduction zone in the 1970s (16) helped to focus earthquake research and monitoring activities during the following decades. While efforts to assess the relative probability of major ruptures in identified seismic gaps became controversial (17–23), being handicapped by consideration of smaller events and the limited information about very large historic earthquakes, almost all very large megathrust earthquakes during the past 50 y have, in fact, been located along subduction zone segments where multiple-decade intervals of prior strain accumulation had occurred (24). Only a handful of recent very large earthquakes have ruptured localized areas where a previous comparable or much larger earthquake was seismically observed, so quantitative comparisons of successive dynamic ruptures remain very limited.

Deployment of geodetic and seismic monitoring instruments in many of the early identified seismic gaps throughout the circum-Pacific region has enhanced the resolution of subsequent faulting processes, revealing heterogeneous coseismic slip on the megathrust fault. New technologies, including global and regional broadband seismograph networks, space-based geodesy (GNSS), satellite interferometry (InSAR), seafloor geodesy (GNSS-a, ocean bottom pressure sensors), seafloor drill hole facilities, and potential field (gravity) measurements, have dramatically improved the ability to quantify long-term strain accumulation and relaxation, as well as short-term coseismic processes, along plate boundaries.

## Results

The following sections consider the fundamental observations concerning the abutting of rupture zones and time-dependent recurrence behavior along the South American plate boundary in the light of recent great earthquakes and the 50 y of subsequent research advances since the initial seismic gap and asperity papers were published. We discuss the spatial and temporal patterns of great earthquake ruptures in the context of updated physical models of the megathrust and identify segments of the plate boundary that appear to have elevated seismic hazard of very large earthquakes within the coming decades. Improved understanding of very large earthquakes on plate boundaries is emerging from observations of many global events (24), but key insights can be captured from consideration of the six recent events along the South American subduction zone. Major observations and lessons learned from these events are summarized below. Detailed discussion and citations for each event are presented in the Supplement.

### 2016 Ecuador.

**Fig. 3.**
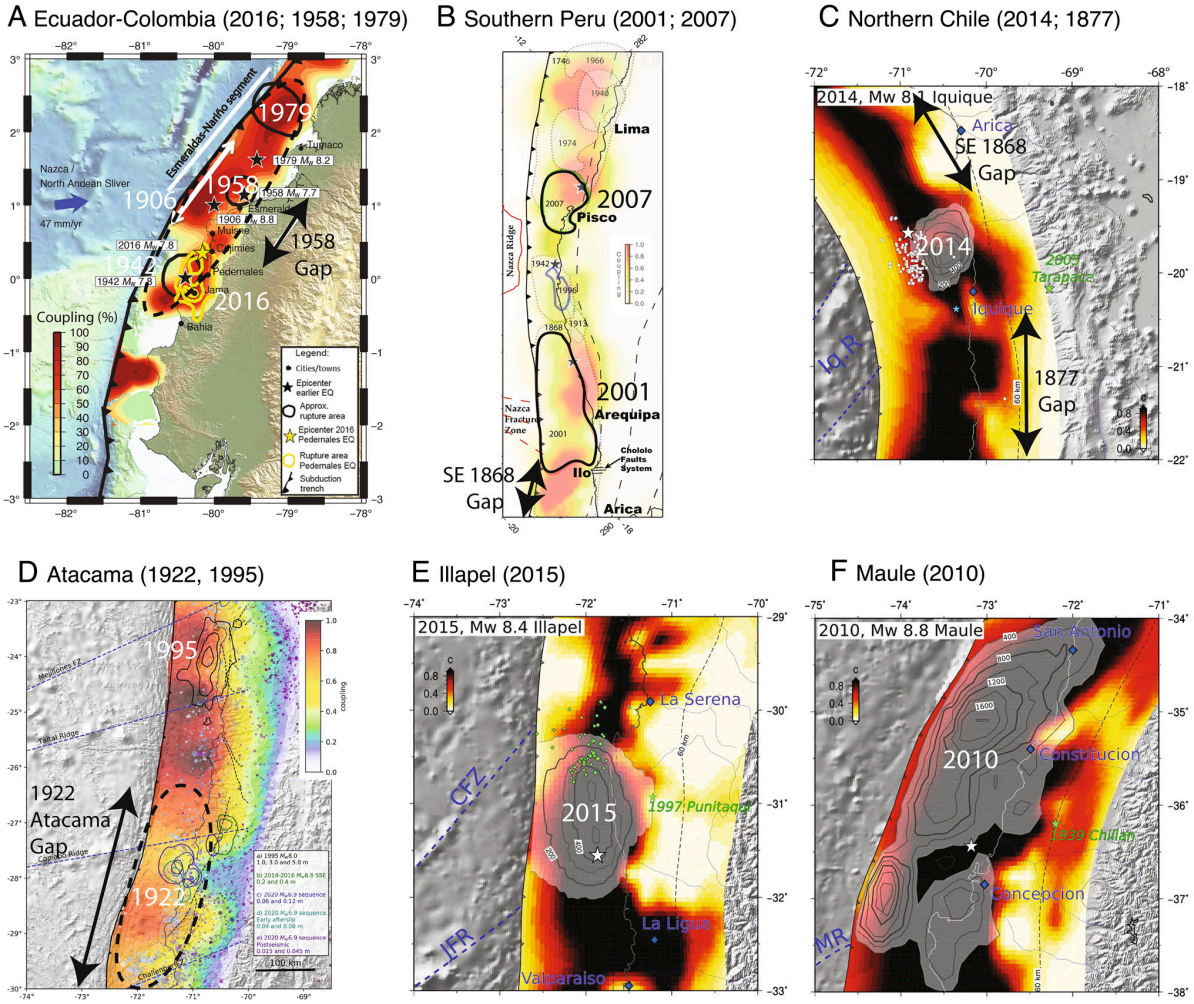
Very large earthquake rupture zone and prior estimates of geodetic plate boundary coupling (darkest reds correspond to 100% slip deficit relative to plate motion) for (*A*) the 2016 Ecuador earthquake and 1906, 1942, 1958, and 1979 ruptures (28); (*B*) the Southern Peru region with the 2007 Pisco and 2001 Arequipa earthquakes (29); (*C*) the 2014 Iquique, Chile, zone, with 1868 Peru to the north and 1877 Chile to the south (9); (*D*) the 1922 Atacama event region with the 1995 Antofagasta earthquake to the north (30); (*E*) the 2015 Illapel earthquake (9); and (*F*) the 2010 Maule, Chile, earthquake (9).

The 16 April 2016 *M_W_* 7.8 Pedernales, Ecuador, earthquake (Figs. 1, 2*A*, and 3*A*) ruptured the down-dip portion of the Colombia/Ecuador seismogenic zone along prior ruptures in 1906 (*M_W_* 8.6) and 1942 (*M_W_* 7.8). Large events to the northeast in 1958 and 1979 fill in most of the 1906 rupture length, demonstrating that great ruptures can intermingle with multiple shorter but still very large events (25). The source region had previously been accumulating moderate slip deficit based on geodetic measurements (26). Comparison of seismic waveforms and magnitudes demonstrate that the 2016 and 1942 events have similar surface wave magnitudes (*M_S_* 7.5), overlapping rupture areas, and an overlapping large-slip patch (Fig. 3*A*) but not identical teleseismic waveforms—indicating that 2016 was a quasirepeat of 1942 (27). This is further discussed in the Supplement. A distribution of slip-weakening patches along strike appears to be characteristic of this region.

### 2007 Pisco, Peru.

The 15 August 2007 (*M_W_* 8.0) Pisco, Peru, earthquake produced substantial shaking damage and a large tsunami on the southern Paracas Peninsula (Figs. 1, 2*B*, and 3*B*). The event is among a sequence of great earthquakes in Central Peru that progressively reruptured the larger 1687 and 1746 zones (Fig. 2*B*) in 1940 (*M_W_* 8.2), 1942 (*M_W_* 8.1), 1966 (*M_W_* 8.1), 1974 (*M_W_* 7.6), and 2007 (*M_W_* 8.0) (31). Geodetic slip deficit had been observed prior to the 2007 rupture (29). The seismic, geodetic, and tsunami data for this event reveal that the rupture involved two or more large-slip patches straddling the peninsula with about a 60-s lag time between the primary subevents (32). The discrete triggering of separated large-slip patches and adjacent up-dip and along-strike afterslip (33) are consistent with the asperity model.

### 2001 Southern Peru.

The 23 June 2001 *M_W_* 8.4 Arequipa (or Camaná), Peru, earthquake and its magnitude 7.6 aftershock on 7 July 2001 to the southeast reruptured the northern two-thirds of the 1868 seismic gap (Figs. 1, 2*B*, and 3*B*). Earthquake intensity and tsunami run-up reports indicate that great events in 1604 and 1868 were larger than those in the overlapping 1582, 1784, and 2001 earthquakes (Fig. 2*B*) (31, 34). Based on analysis of seismic, geodetic, and tsunami data, the earthquake broke two spatially offset asperities: the first in the northwest of the rupture zone and the second, centrally located asperity being much larger and releasing most of the total seismic moment (29, 32, 35). Rupture appears to have extended across the megathrust to near the trench.

### 2014 Iquique, Chile.

The 1 April 2014 *M_W_* 8.1 Iquique, Chile, earthquake and its large *M_W_* 7.7 aftershock on 3 April 2014 to the south ruptured a rather compact area of the northern Chile central megathrust from 19.3°S to 20.7°S (Figs. 1, 2*C*, and 3*C*). The rupture was preceded by months of slowly migrating foreshock activity located up-dip of the eventual mainshock, indicating along-dip variation in frictional properties of the megathrust (36, 37). The large-slip zone (~2 to 7 m) for the 2014 mainshock extends only about 70 km along strike and 50 km along dip, with finite-slip models being well resolved by seismic, geodetic, and tsunami observations (38, 39). The concentrated mainshock slip, with adjacent down-dip slow deformation and afterslip, is consistent with the asperity model, and several prior historical earthquakes have occurred in this region of northernmost Chile over the past few centuries (Fig. 2*C*), so persistence of localized velocity-weakening properties is viable. The event struck in an area of large slip deficit inferred from geodesy that extends along northern Chile from 18°S to 25°S, with a low-coupling zone near 21°S (40). Many estimates of the 1877 rupture extent span this region (41, 42), so early interpretations viewed the 2014 event as a partial rupture of the 1877 zone akin to the events along Ecuador–Colombia. However, based on detailed reinterpretation of intensity observations for 1877, the 2014 Iquique event appears to have ruptured within the megathrust region south of Arica and north of Iquique that lies between large-slip regions of the great 1868 Peru and 1877 Chile earthquakes (43) (Fig. 3*C*).

### 2015 Illapel, Chile.

The 16 September 2015 *M_W_* 8.3 Illapel, Chile, earthquake ruptured ~170 km along the plate boundary megathrust in central Chile from 30°S to 31.8°S (Figs. 1 and 3*E*). This event struck in the same region as events in 1943, 1880, and 1730 (Figs. 2*C* and 3*E*) (18, 44). The 2015 Illapel earthquake is of particular note because rapid seismic magnitude estimation of the event prompted a tsunami warning and evacuation notifications within 8 to 11 min of the origin time, resulting in large-scale evacuation along the Chile coast (45). Seismic, geodetic, and tsunami waveform analyses of the 2015 Illapel earthquake indicate concentrations of ~3-m coseismic slip below the coast and a large patch with up to ~10-m slip at shallow depths (46–48). Studies with the best offshore resolution are consistent with the large-slip patch having extended up-dip to near the trench. Geodetic measurements prior to the event indicate that there was strong megathrust coupling in the region of large slip, particularly south of 31°S, although resolution of coupling out to the trench is very low (49, 50), and afterslip expanded both northward and southward from the large-slip zone (51). The prior 1943 *M_W_* 7.9 event has a single pulse of moment release at depths <35 km but has a smaller seismic moment estimate and simpler waveforms that indicate that it did not rupture the shallow portion of the megathrust (50). Local and far-field tsunami heights for the 2015 event are significantly higher than those in 1943. Overall, the 2015 event is not a simple repeat of the 1943 event and likely had much more slip at shallow depth (45).

### 2010 Maule, Chile.

The 27 February 2010 Maule (*M_W_* 8.8) earthquake ruptured the plate boundary offshore of central Chile between 34°S and 38.5°S (Figs. 1–3*F*). The coseismic slip of this event has been determined by analysis of seismic, geodetic, and tsunami observations. Patchy coseismic slip is distributed over a region 460 km long and 100 km wide between the depths of 15 and 40 km. Two large-slip asperity regions are resolved along the megathrust: one extending from 34°S to 36°S (with up to 20-m slip) and the other from 37°S to 38°S (with up to 10-m slip). Joint inversions with accurately modeled tsunami observations find that the large-slip patches include slip of 5 to 8 m all the way to the trench (52, 53). Geodetic measurements had resolved accumulating slip deficit prior to the rupture along the entire rupture area, with moderate reduction near 35°S (54), but the patchy slip distribution only loosely conforms to the variable locking distribution (55). Afterslip extends along the length of the rupture primarily down-dip and between the two large coseismic slip patches (56). Conventional seismic gap ideas with strong segmentation do not characterize this region well, but the Reid strain renewal concept in conjunction with a distribution of persistent asperities along the megathrust reconciles the historical behavior.

## Discussion

**Fig. 4.**
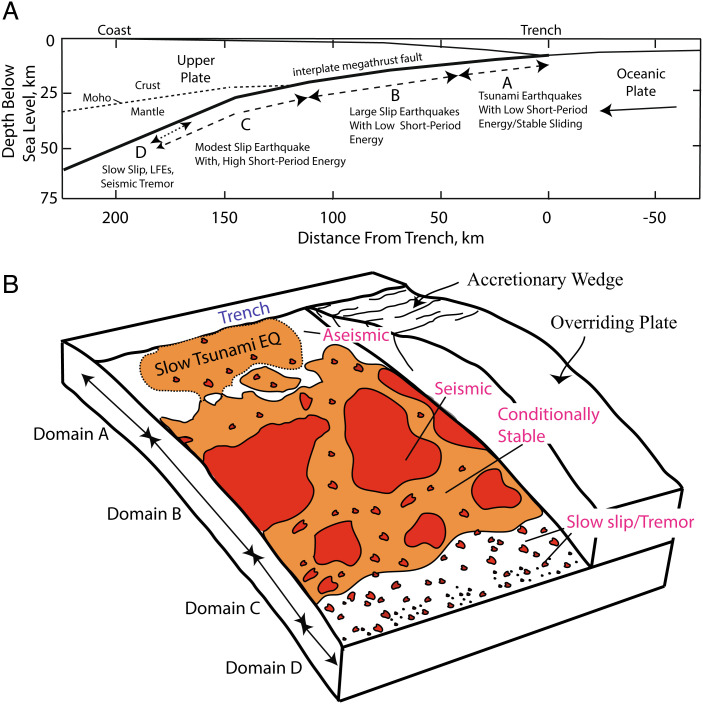
An updated representation of the asperity model (24). (*A*) Schematic cross-section indicating four depth-varying domains of megathrust rupture characteristics: A – near-trench domain where tsunami earthquakes or anelastic deformation and stable sliding occur; B – central megathrust domain where large slip occurs with minor short-period seismic radiation; C – down-dip domain where moderate slip occurs with significant coherent short-period seismic radiation; D – transitional domain, only present in some areas, typically with a young subducting plate, where slow slip events, low-frequency earthquakes, and seismic tremor can occur. At yet greater depths, the megathrust slides stably or with episodic slow slip or plastic deformation that does not generate earthquakes. (*B*) Cutaway schematic characterization of the megathrust frictional environment related to Domains A, B, C, and D defined in (*A*). Regions of unstable frictional sliding (asperities) are red regions labeled “seismic.” Regions of aseismic stable or episodic slow sliding are white regions labeled “aseismic.” Orange areas are conditional stability regions, which displace aseismically except when accelerated by failure of adjacent seismic patches. Domain A is at shallow depth where low-rigidity sediments and pore fluids cause very slow rupture expansion even if large displacements occur in tsunami earthquakes. Domain B has large, relatively uniform regions of stable sliding that can have large slip but generate modest amounts of short-period radiation upon failure. Domain C has patchy, smaller-scale regions of stable sliding surrounded by conditionally stable areas. When these areas fail, coherent short-period radiation is produced. Small, isolated patches may behave as repeaters when quasistatic sliding of surrounding regions regularly load them to failure. Domain D is dominated by aseismic sliding, but many small unstable patches can rupture in seismic tremor when slow slip events occur.

The quantification of interseismic, coseismic, and postseismic deformation for the six very large earthquakes along the South American subduction zone in the past 21 y described above provides insight into updated conceptual/observational seismic gap and asperity models. The intuitive concept of strain accumulation and release in the Reid renewal cycle continues to underlie validity of the seismic gap idea for very large earthquake occurrence, but strict segmentation of the plate boundary is not defined by recent rupture zones. Early estimates of the lateral extent of large ruptures relied heavily on aftershock zones as well as MMI VIII damage and tsunami reports. Recent, well-documented earthquakes help to calibrate these older descriptions (7). Coseismic slip heterogeneity and nonuniform slip deficit accumulation from seismic and geodetic inversions continue to be well accounted for by the asperity model, but evaluating persistence of these regions of slip-weakening properties is complicated by repeated very large earthquakes having variable slip both along dip and along strike. Representations of the asperity model have progressively added complexity to reflect along-dip variations and complexity of individual sequences (Fig. 4) (24, 57–59), and such models have been invoked in many earthquake studies. Along-dip variations are now recognized as particularly important, with the megathrust shallower than 15 km (Domain A) potentially having strain accumulation that results in tsunami earthquakes or enhances ruptures that initiate deeper. Between 15 and 35 km (Domain B), the megathrust has discrete slip-weakening patches that are patchy and surrounded by slip-strengthening zones; the larger patches fail in very large earthquakes and may cascade to produce great earthquakes that span longer stretches of the boundary. Domain C extends from 35 to 50 km and has reduced size asperities and increasing aseismic component, but damaging earthquakes can still result as they tend to be below the coast. This region also produces stronger short-period radiation during very large earthquakes.

Bathymetric features on the subducting plate, notably the Chile Rise, Challenger Fracture Zone, Juan Fernandez Ridge, Nazca Ridge, Medaña Fracture Zone, and Carnegie Ridge, appear to act as persistent barriers to rupture along South America, defining major megathrust segments (3, 60). Finer-scale segmentation is controlled by asperity distributions on the megathrust, but only a few examples (1942/2016 Ecuador and 1943/2015 Illapel) of repeated ruptures with seismic recordings are available to evaluate the persistence of asperities through the seismic cycle. Megathrust ruptures that span the entire width of the plate interface (Domains A+B+C), termed “Breakthrough Ruptures” (61), are proposed to “reset” the seismic cycle and are distinct from those events confined to deeper portions of the interface (Domain B or C only). Along the South American plate boundary, one can identify multiple Breakthrough Ruptures, including the 1575 and 1960 S. Chile, 1730 Valparaíso, 1819/1922 Atacama, 1877 N. Chile, 1604/1868 S. Peru, 1746 Central Peru, and 1906 Colombia–Ecuador events. From two to four events have reruptured most of the same regions in smaller, nonoverlapping events, giving rise to the space–time irregularity evident in Fig. 2 but still allowing regions of significant strain accumulation and potential for future events to be identified.

If we view seismic gaps in areas with prior very large earthquakes and/or current day slip deficit accumulation as regions with patchy asperities that must accumulate sufficient stress and strain to fail, one can generally infer relative seismic hazard based on historical and geodetic observations. Essentially, the updated asperity representation shown in Fig. 4 captures the essence of the asperity, seismic gap, and frictional heterogeneity perspectives, with the behavior of the larger asperities being emphasized here. With the 2016 Ecuador, 2007 and 2001 Peru, and the 2010 Maule events all involving coseismic rupture of at least two large asperities, and the 2001 Peru and 2014 Iquique, Chile, events having very large aftershocks along strike, the patchy nature of the megathrust asperity distribution has been clearly manifested in the recent South American events. The relatively uniform but modulated geodetic coupling on the megathrust along the South America coastline, with patchy ruptures and afterslip distributions for the recent very large events, provides further support for this conceptual model. However, time predictability remains elusive given the experience that some events involve cascades of several asperities failing together to make a great earthquake, some events likely have incomplete stress release due to lateral buttressing by adjacent regions that do not fail, and shallow megathrust failures may or may not accompany deeper megathrust failures. The recent events demonstrate this full range of behavior. Anticipating the size and timing of future events is thus highly uncertain, but as for the recent events, one can generally anticipate where large events are likely to occur.

With these perspectives in mind, we identify four regions of particular interest for future large earthquake occurrence.

• *Ecuador/Colombia:* Esmeraldas (~1°N)

The region just north of the 2016 *M_W_* 7.8 Ecuador rupture (Figs. 1, 2*A*, and 3*A*) last ruptured with a comparable size event in 1958 (*M_W_* 7.6). Viewing the deeper megathrust region as having several large asperities distributed along strike, the 2016 failure has increased driving stress on the 1958 zone, which already has 64 y of possible strain accumulation, exceeding that between the 1906 and 1958 events. Aftershock activity for the 2016 event has concentrated offshore and along the southwestern portion of the 1958 zone. Localized strong geodetic coupling in the 1958 rupture zone adds to the earthquake potential in this region.

• *Southeasternmost Peru:* Arica (~18 to 19°S)

The 1604 and 1868 MMI VIII isoseismal zones both extend farther southeast toward Arica, Chile, than the 2001 rupture (Figs. 1, 2*B*, and 3*B*), indicating that the southeasternmost portion of the Peru plate boundary has remained unbroken for 154 y (34, 62). Geodetic slip deficit accumulation in the area is high (~63 mm/y) indicating that as much as ~10 m of slip may have accumulated in the region since 1868, with potential seismic moment equivalent to an *M_W_* 8.4 event. It is unclear why the 2001 event failed to rupture into this region, but there is evidence for prior smaller events that ruptured just this region in 1833 and 1715 (Fig. 2*B*).

• *Northern Chile:* Loa (~21 to 23°S)

The Loa segment between Iquique and Antofagasta corresponds to the large-slip region of the great 1877 Arica earthquake based on intensity reports (41, 43) and is bounded to the north by the 2014 *M_W_* 8.2 Iquique earthquake and to the south by the 1995 *M_W_* 8.0 Antofagasta rupture (Figs. 1, 2*C*, and 3*C*). The Loa segment exhibits high geodetic coupling along its entire length (Fig. 3*C*), and the area between 20° and 21°S has had little to no seismic activity during the last century (39). The rate of slip deficit accumulation in the area (~55 mm/y) (63) indicates that as much as ~8 m of slip has accumulated in the region since 1877, with potential seismic moment equivalent to an *M_W_* 8.4 event. Rupture of the shallow megathrust up-dip of the 2007 rupture zone as part of this event is viable.

• *Northern Chile:* Vallenar/Atacama (~26 to 29.5°S)

This region last ruptured in the great *M_w_* 8.6 Atacama earthquake of 10 November 1922 and is bounded to the north by the 1995 *M_W_* 8.0 Antofagasta rupture and to the south by the 2015 Illapel *M_W_* 8.3 earthquake (Figs. 1, 2*C*, and 3*D*). The northern region of the 1922 rupture zone, from 26°S to 27°S, has experienced relatively frequent large ruptures, in 1796 (M ~ 7.5), 1819 (M ~ 8.5), 1859 (M ~ 7.5), 1918 (M ~ 7 to 7.5), 1922, 1946, and 1983 (*M_W_* 7.6), while the southern region from 27°S to 29.5°S appears to have ruptured only in 1819 and 1922 (7, 8, 64) (Fig. 2*C*). The 1922 event likely exhibited bilateral rupture (65) and a complex slip distribution involving the rupture of three separate asperities, seemingly consistent with eyewitness accounts (44). The prior rupture in 1819 involved a sequence of three events on April 3, 4, and 11 (8). The very large earthquake pairs in 1796/1819 and 1918/1922 have been suggested to represent the primary plate boundary ruptures for the Vallenar/Atacama segment, indicating a repeat time for this segment of the Chilean subduction zones of on the order of a century. Geodetic surveys provide a clear mapping of heterogeneous interseismic coupling along the 1922 rupture zone with high coupling at both shallow (8 to 15 km) and intermediate (15 to 35 km) depths (30, 49, 63) (Fig. 3*D*). The southern boundary of the 1922 rupture, near La Serena (30°S), is coincident with the intersection of the Challenger Fracture Zone, and the local low geodetic coupling is proposed to act as a persistent barrier between great earthquake rupture in the Atacama and south-central Chile segments (66). For an estimated slip deficit rate of ~50 mm/y (63), ~5 m of slip may have accumulated during the last 100 y comparable with an *M_W_* 8.3 earthquake.

Looking forward, sustained operation or new deployment of dense networks of seismic, onshore and offshore geodetic, and tsunami sensors is essential to making sufficient observations of the deformation process in these four regions that will inevitably culminate in future very large earthquakes. Large-scale space–time patterns of regional seismicity may help to identify regions approaching their limiting strain accumulation (61, 67). Of course, large events can also occur in regions where strain accumulation is thought to be modest; the 2016 *M_W_* 7.6 earthquake in the 1960 rupture zone (Fig. 2*D*) is one such example. Imprecise knowledge of strain release in historical events limits the ability to anticipate such behavior. But this does not eliminate the value of concentrating observational effort on regions that likely will experience future very large events, given the success that this strategy has achieved for recent South American earthquakes.

## Materials and Methods

Earthquake rupture source dimensions and, for recent events, coseismic slip distributions for ruptures along the South American subduction zone were extracted from the literature. This information is incorporated into Figs. 1 and 2, which document the very large earthquake history dating back to 1500. The rupture lengths for historic events are largely based on documented ground shaking and damage patterns, with information being available for very large events for regional and far-field tsunami inundations. The history of events prior to 1900 is nonuniform along the coast over the past 500 y as it depends on European settlements and archives. In limited regions, sedimentological observations document great events over several millennia. Details of many of the earthquakes extracted from geological, seismological, geodetic, and tsunami observations are discussed and cited in the supplement, with a focus on six recent large events that have been particularly well studied. These observations of the history of large earthquakes along the subduction zone are considered in the context of seismic gap and seismic asperity conceptual models to understand the variation in earthquake ruptures along localized subduction zone segments and to highlight regions with large strain accumulation where future great earthquakes are likely to occur and where geophysical instrumentation can be deployed to capture the later stages of the earthquake cycle culminating in the large events to come.

## Supplementary Material

Appendix 01 (PDF)

## Data Availability

All study data are included in the article and/or *SI Appendix*. No new data were generated in this study.
