# Supplementary material for: Updated concepts of seismic gaps and asperities to assess great earthquake hazard along South America
Source: Proc Natl Acad Sci U S A. 2022 Dec 13;119(51):e2216843119. doi: 10.1073/pnas.2216843119 (PMC9907104; doi:10.1073/pnas.2216843119)
Supplement: Supplementary file 1 — Appendix 01 (PDF) [file pnas.2216843119.sapp.pdf]

Updated concepts of seismic gaps and asperities to assess great earthquake hazard along South America

Thorne Lay[a,*] and Stuart P. Nishenko

Supporting Information

Ecuador-Colombia

The Ecuador-Colombia plate boundary from 4°N to 3°S (Figure 1) involves oblique underthrusting of the Nazca plate at ~4.6 cm/yr below the North Andean Sliver, a fragment of the South American plate (1-3). The broad Carnegie Ridge on the incoming oceanic plate intersects the subduction zone from 0.5°N to 2.0°S (4). Modeling of interseismic geodetic strain around the megathrust requires accounting for the movement of the sliver relative to stable South America along with any distributed deformation in the northern Andes. Doing so indicates heterogeneous locking of the plate interface from 3°N to 3°S, with relatively uniform >40% locking north of 0.5°S and an isolated patch below La Plata Island from 1°S to 1.5°S where a slow-slip event occurred in 2010 and no large (M_W >7) earthquake has been recorded (2; 5-7).

The history of very large megathrust earthquakes along this region is relatively short (Figure 2a). The 1906 M_w 8.6 Columbia/Ecuador earthquake is the largest known event. It had an estimated rupture length of ~500 km, based on macroseismic data (8), and produced significant local and Pacific wide tsunami (M_T 8.7, 9). Historic information on earlier events provide only indirect evidence for recurrence times in that no event comparable to 1906 is recorded in the historic catalog from 1575 to 1915 (331 years) (10). Earthquake triggered turbidites collected on the continental slope offshore of Esmeraldas River indicate that one or two earthquakes comparable in size to the 1906 event occurred ~600 years ago (11).

A series of great earthquakes re-ruptured the near coastal portion of the 1906 Colombia/Ecuador zone within 36 (1942, M_S 7.5), 52 (1958, M_S 7.3) and 73 (1979, M_S 7.7) years of 1906 (12). An M_W 7.1 event in 1998 ruptured the southernmost portion of the 1906 zone southwest of the 1942 rupture (2). The aftershock zones of these ruptures abut without overlap within the larger 1906 rupture zone (13). Analysis of seismic waveforms (14) and GPS data (2) has identified discreet asperities associated with the 1958 and 1979 ruptures. Kanamori and McNally (12) note that the cumulative seismic moment of the 1942, 1958 and 1979 earthquakes based on aftershock zone area is considerably less (~1/5) than the seismic moment of 1906. This discrepancy reduces to ~1/3 based on direct waveform comparisons (14).

The re-rupture of the 1942 Pedernales, Ecuador segment in 2016 (M_W 7.8, M_S 7.5) presents an opportunity to examine persistent heterogeneous frictional properties of the Colombia-Ecuador megathrust, and may indicate the onset of a new earthquake cycle along the Colombia-Ecuador region.

2016 Ecuador

The 16 April 2016 M_W 7.8 Pedernales, Ecuador earthquake (Figures 1, 2a, 3a) ruptured the down-dip portion of the Colombia/Ecuador seismogenic zone along prior ruptures in 1906 (M_W 8.6) and 1942 (M_W 7.8). The source region had previously been accumulating moderate slip deficit based on geodetic measurements (2), with larger slip deficit accumulating in the adjacent regions of non-overlapping aftershock zones of the 1958 (M_W 7.6) and 1979 (M_W 8.1) ruptures, extending along the 1906 zone. Chlieh

et al. (2) estimated characteristic earthquake recurrence times for asperities associated with 1942, 1958 and 1979 events of ~140±30, 90±20 and 153±80 years, respectively, significantly exceeding their actual intervals since 1906 (36, 52, and 73 years). After 74 years, the 1942 region re-ruptured in the 2016 event.

Available high-rate GPS, broadband teleseismic, InSAR, and tsunami data resolve the rupture of two large-slip patches in 2016 with peak slip of ~ 2-6 m and an average slip ~ 2 m (6, 15-18). Comparison of seismic waveforms and magnitudes demonstrate that the 2016 and 1942 events have similar surface wave magnitudes (M_S 7.5), overlapping rupture areas, and an overlapping large-slip patch, but not identical teleseismic waveforms – indicating that 2016 was a quasi-repeat of 1942 (15, 19). While the average slip in 2016 is consistent with the plausible slip deficit accumulation of 3.5 m since 1942, given the ~4.7 cm/yr convergence rate (15), localized peak slip estimates of 5-6 m exceed the expected slip deficit (6), indicating that significant residual slip deficit persisted after the 1942 event in the localized region of peak slip in 2016. Nocquet et al. (6) also infer excessive moment release in the 1958 and 1979 events relative to slip deficits accumulated since 1906. Noting the lack of historic large earthquakes in the region (Figure 2a), they propose that the Ecuador-Colombia region has been experiencing a supercycle of large events over the past century. Yoshimoto et al. (18) invert for the tsunami source of 1906, finding large-slip on the shallow megathrust, up-dip of the large-slip zones in 2016, 1942, 1958 and 1979, complicating assessment of strain budget for localized regions of the megathrust.

PERU

The Peru seismic zone, extending from 3°S to ~19°S, has the most pronounced variability in very large megathrust faulting history of the entire South American seismogenic zone (Figures 1, 2b). The Northern Peru segment from 3°S to 10°S is bounded by the Grihalva Ridge to the north and the Mendaña Fracture zone to the south. Geodetic measurements in the region (1, 20-21) indicate that the plate boundary is not accumulating significant slip deficit along the 800-km-long segment other than in localized shallow (<20 km deep), poorly resolved patches near 3°S-4°S and 7°S-8°S. The area near Chimbote (~9°S) has experienced infrequent large earthquakes (Figure 2b), the largest being M_W ~ 7.7-8 in 1619 (8; 21-23). That event destroyed the town of Trujillo and macroseismic reports indicate that damage extended over 100-150 km. The most recent large events along Northern Peru are the 1960 M_W 7.6 and 1996 M_W 7.5 earthquakes located on the shallow megathrust (Figure 1, 2b), which have both been characterized as tsunami earthquakes due to having weak radiation of short-period seismic energy, low rupture velocity and long rupture durations (24-27). Future occurrence of very large earthquakes in this region is very difficult to anticipate based on the coupling and historical records.

The seismic record for central and southern coastal Peru (Figure 2b) is considered complete for earthquakes of M > ~7.6 for more than 450 yr (21-23; 28). The Central Peru segment from 10°S to 14.5°S is bounded by the Mendaña Fracture Zone to the north and the Nazca Ridge to the south. The subducted Nazca plate in central Peru is characterized by flat, low angle subduction and a lack of active volcanism. Dorbath et al. (23) describe the seismic activity in Central Peru as being complex due to the irregularity of rupture lengths, locations of epicentral zones, and timing. Two earthquakes stand out in the historic record, not only for their size but also for the length of time of seismic quiescence following their occurrence. The 1687 M_W 8.4 Ica earthquake ruptured the southern half of the central Peru segment with an estimated rupture length of 350 km and produced a damaging local tsunami with a height of 5 to 10 m (M_T 8.5-8.4, 9). The 1746 M_W 8.6 Lima, Peru earthquake ruptured the northern 350 km of the central Peru segment 59 years later with long overlap of the 1687 zone and produced a local tsunami of 15 to 24 m height (M_T 9 – 9.2, 9). The 1746 event ranks as the largest Peruvian earthquake during the last 450 years (23) and coupled with the earlier 1687 earthquake (the slip distributions are not known in detail) may represent a so-called "Breakthrough Event" (29) that ruptured the entire Central Peru segment (30). Following these two events, a period of seismic quiescence for great earthquakes along much of Central Peru lasted nearly 200 years (23).

A renewed period of earthquake activity spanning Central Peru started in 1940. A series of great earthquakes progressively re-ruptured portions of the 1687 and 1746 zones in 1940 (M_W 8.2), 1966 (M_W 8.1), 1974 (M_W 8.1) and 2007 (M_W 8.0) (8; 22-23; 30-31). These recent events occurred at intermediate depths along the megathrust (15 to 35 km), and exhibit non-overlapping rupture zones (Figures 1, 2b) consistent with the seismic gap and asperity concepts. Waveform analysis of these events (18, 31-35) identified one to three concentrated large-slip zones, or asperities, for each event. The events produced minor local tsunamis ranging from 1.6 to 3 m in height that were significantly less than those reported for 1687 and 1746. This is similar to the Ecuador-Colombia region. Chlieh et al. (36) estimate that the recent set of events account for less than half of the estimated seismic moment release in 1746, leaving a deficit that could produce an M_W 8.5-8.7 event. The 2007 rupture struck the southeastern end of this region, which had not had a very large earthquake since 1746.

2007 Pisco, Peru

The 15 August 2007 (M_W 8.0) Pisco, Peru earthquake produced substantial shaking damage and a large tsunami on the southern Paracas peninsula (Figures 1, 2b, 3b), northwest of the intersection of the Nazca Ridge with the Peru Trench. The seismic, geodetic and tsunami data for this event reveal that the rupture involved two or more large-slip patches straddling the peninsula with about a 60 s lag time between the primary subevents (e.g., 20; 34-43). Maximum slip was up to about 8 m and geodetic slip deficit had been observed prior to the rupture (36).

The discrete ruptures during this event, with two main separated asperities experiencing triggering interaction and adjacent up-dip and along-strike afterslip with seismic moment equal to 14% of the co-seismic moment (20) are consistent with the asperity model, but this type of multi-asperity delayed rupture presents great challenges to early warning procedures that attempt to characterize imminent seismic and tsunami hazards from the early energy release or ground deformation (34). Longer term, interseismic coupling models indicate as much as 50-70% aseismic slip in this region and are consistent with return times of 250 years or greater (i.e., 2007 – 1687 = 261 yrs), in this region just north of where the Nazca ridge intersects the subduction zone (20). Given the lack of seismic recordings of prior events striking the recent rupture zones, we cannot assess persistence of asperities in Central Peru.

The Southern Peru segment extends from 14.5°S, where the Nazca Ridge intersects the trench, to ~19°S, near the Chilean border and Arica. Great earthquakes have occurred relatively frequently in Southern Peru (22-23; 44) during the last 500+ years (Figure 2b). Great ruptures spanning this segment struck in 1604 and 1868, with pairs of very large events (1687/1715 and 1784/1833) also covering most of the length. The region in the north near the city of Nazca had several large ruptures in 1913, 1942 (M_W 8.1) and 1996, with the latter two being partially overlapping complex ruptures along the southern flank of the Nazca Ridge intersection (19; 45).

2001 Southern Peru

The 23 June 2001 M_W 8.4 Arequipa (or Camaná), Peru earthquake and its magnitude 7.6 aftershock on 7 July 2001 to the southeast, re-ruptured the northern two-thirds of the 1868 seismic gap (Figures 1, 2b, 3b). Based on analysis of seismic, geodetic and tsunami data, the earthquake broke two spatially offset asperities, the first in the northwest of the rupture zone and the second, centrally located asperity being much larger and releasing most of the total seismic moment (34; 36-37; 46-49). Rupture appears to have extended across the megathrust to near the trench (34; 36), unlike the 2007 Pisco and 2016 Ecuador events.

Earthquake intensity and tsunami runup reports indicate that great events in 1604 and 1868 (10-15 m and 14 m peak tsunami runup, respectively) were larger than the overlapping 1582, 1784, and 2001

earthquakes (1-2 m, 2-4 m, and 8.8 m peak tsunami runup, respectively) (Figure 2b) (23; 46). Lacking seismic recordings it is not possible to compare details of the ruptures or to assess persistence of asperities, but the repeated occurrence of great earthquakes with overlapping ruptures is consistent with the basic seismic gap concept, with frictional heterogeneity resulting in smaller slip patches adjacent to a large central asperity. The 1604 and 1868 MMI VIII isoseismal zones both extend farther southeast toward Arica, Chile than the 2001 ruptures, indicating that the southeasternmost portion of the Peru plate boundary has remained unbroken for 154 years (46; 50-51). Geodetic slip deficit accumulation in the area is high (~63 mm/yr) indicating that as much as ~10 m of slip may have accumulated in the region since 1868, with potential seismic moment equivalent to an M_W 8.4 event. It is unclear why the 2001 event failed to rupture into this region, but there is evidence for prior smaller events that ruptured just this region in 1833 and 1715 (Figure 2b).

CHILE

Northern Chile

The Northern Chile region extending from 19°S to 26°S has a limited very large earthquake history, dominated by the great 1877 (M_W 8.5-8.8) and 1995 Antofagasta (M_W 8.0) earthquakes (Figures 1, 2c) (52). Large events for which there is some information struck northernmost Chile in 1615, 1768 and 1786, in the vicinity of the recent 2014 Iquique event (44). There is marine evidence for slumping near 23°S occurring between 1754 and 1789 (53), indicating that the 1768 and/or 1786 ruptures may have extended along the entire 1877 zone. Marine evidence near 23°S and boulder fields on the Atacama coast also indicate a predecessor event overlapping the 1877 event around 1429 ± 20 (53, 54), coincident with Japanese tsunami records of a distant event on 7 September 1420 (55). Geologic and archeological provide evidence for a giant (M ~ 9.5) earthquake in this region at ~3800 years ago (56) that may have also affected the Northern Chile and Atacama Desert region from 21° to 27° S.

2014 Iquique, Chile

The 1 April 2014 Mw 8.1 Iquique, Chile earthquake and its large M_W 7.7 aftershock on 3 April 2014 to the south ruptured a rather compact area of the northern Chile central megathrust from 19.3°S to 20.7°S (Figures 1, 2c, 3c). The large-slip zone (~2-7 m) for the 2014 mainshock is unusually concentrated for a great earthquake, extending only about 70 km along strike and 50 km along-dip, with finite-slip models being well resolved by seismic, geodetic, and tsunami observations (57-64). The rupture was preceded by months of slowly migrating foreshock activity located up-dip of the eventual mainshock, indicating along-dip variation in frictional properties of the megathrust (59; 65-71)

The concentrated mainshock slip, with adjacent down-dip slow deformation and afterslip (71; 72) is consistent with the asperity model, and several prior historical earthquakes have occurred in this region of northernmost Chile over the past few centuries (Figure 2c), so persistence of localized velocity weakening properties is viable. The event struck in an area of large slip deficit inferred from geodesy that extends along northern Chile from 18°S to 25°S, with a low coupling zone near 21°S (36; 72-74), although the coupling estimates depend strongly on assumptions of upper plate (central Andes) distributed deformation. Many estimates of the 1877 rupture extent span this region (e.g., 44; 75), so early interpretations viewed the 2014 event as a partial rupture of the 1877 zone akin to the events along Ecuador-Colombia. However, based on detailed reinterpretation of intensity observations for 1877, the 2014 Iquique event, rupturing the megathrust region south of Arica and north of Iquique lies between large-slip regions of the great 1868 and 1877 earthquakes (76) (Figure 3c). The 1877 slip zone may or may or may not have overlapped the 2014 event, and while it extends along the 2007 Tocopilla event at its southern end (Figure 2c), the latter event was concentrated down-dip in Domain C and did not rupture the shallow megathrust (36; 77-80).

1995 Antofagasta

The 30 July 1995 M_W 8.0 Antofagasta earthquake ruptured south of the 1877 earthquake gap from 23.3°S to 25°S (Figures 1, 2c, 3d). Analysis of seismic, geodetic, and tsunami data indicate that the rupture began near the Mejillones peninsula and expanded southward with predominantly unilateral slip (81-88), to the vicinity of the 1966 (M_S 7.8) Tal-Tal earthquake at its southern end. Long-period directivity indicates a rupture velocity of 3.0-3.2 km/s and rupture duration of 60-68 s (85). The finite-fault studies resolve slip beneath the coastal area in the central megathrust (Domain B of Figure 4), with some along-strike variability that may be due to prior stress relaxation in 1987 (M_W 7.5) and 1988 (M_W 7.2) ruptures and a 1998 (M_W 7.0) aftershock in the deeper portion of the megathrust (Domain C of Figure 4) (87; 89). The rupture south of 24.3°S appears to have modest slip that extends to near the trench (Domain A of Figure 4) based on strong excitation of *pwP* arrivals (27; 90), and there is some indication of this in finite-fault modeling, although such models lack resolution of slip near the trench (88).

North-Central Chile - Atacama

Seismic waveform modeling (91) indicates rupture of 3 sub-events during the 1922 Atacama earthquake, consistent with eyewitness accounts of feeling three distinct shocks within the first few minutes. The prior great rupture in 1819 involved a sequence of three events on April 3, 4 and 11, as well (92). As seen in Figure 3d, a line of seamounts intersects the Chile trench near 27°S, in the northern portion of the 1922 Atacama earthquake rupture zone which has had repeated smaller events in 1851, 1859, 1918, 1946 and 1983 (Figure 2c). The seamounts are spaced ~ 100 to 150 km apart and are ~25 km in diameter. Each seamount or asperity could accumulate a slip deficit of 6 to 7 m per century, equivalent to an M 7+ earthquake. While the seismic moments of subevents in 1922 are not well constrained (91; 93), the rough seafloor bathymetry may account for some of the rupture complexity. Evidence for prior great ruptures from paleotsunami run-up along the Atacama include the 1429 ± 20 event (53; 54) discussed above, along with 1267 ± 85? and 964 ± 32? segment-spanning events (94).

Central Chile

The Illapel region (30°S-32°S) (Figures 1, 3e) is a highly coupled segment of central Chile bounded by the Challenger Fracture Zone (CFZ) to the north and the Juan Fernandez Ridge (JFR) to the south (95-97). The CFZ intersects the Chile Trench near the southern end of the 1922 Atacama earthquake and at the estimated northern end of the great 1730 Valparaíso earthquake, suggesting persistent segmentation. The Illapel segment exhibits complexity of very large earthquake rupture as it ruptured in the northern ~1/3 of the great 1730 M_W ~ 9 Valparaíso earthquake as well as in a series of smaller overlapping events in 1880 (M_W 8.3), 1943 (M_W 7.9), and 2015 (M_W *8.3*). The latter set of ruptures may possibly involve a persistent asperity on the central megathrust, but with variable amounts of shallow coseismic slip near the trench. There is no clear data on great events prior to 1730, extending south to Constitución.

2015 Illapel, Chile

The 16 September 2015 M_W 8.3 Illapel, Chile earthquake ruptured ~170 km along the plate boundary megathrust in Central Chile from 30°S to 31.6° S (Figure 1). This event struck in the same region as events in 1943, 1880, and 1730 (Figures 2c, 3e) (8; 91; 98-99). The 2015 Illapel earthquake is of particular note because rapid seismic magnitude estimation of the event prompted a tsunami warning and evacuation notifications within 8 to 11 min of the origin time, resulting in large-scale evacuation along the Chile coast (100). Seismic, geodetic, and tsunami waveform analyses of the 2015 Illapel earthquake indicate concentrations of ~3 m co-seismic slip below the coast and an large patch with up to ~10 m slip at shallow depths (94; 100-113). Studies with the best off-shore resolution (including careful modeling of tsunami arrivals) are consistent with the large-slip patch having extended up-dip to near the trench.

Geodetic measurements prior to the event indicate that there was strong megathrust coupling in the region of large-slip, particularly south of 31°S, although resolution of coupling out to the trench is very low (106; 114-115), and afterslip expanded both northward and southward from the large-slip zone (110; 116-118).

Similar to the 2016 Ecuador earthquake, comparisons can be made with details of the prior very large rupture in 1943. The 1943 M_W 7.9 event has a single pulse of large moment rate at depths < 35 km but has a much smaller seismic moment estimate and simpler waveforms that indicate that it did not rupture the shallow portion of the megathrust (91; 101). Local tsunami heights for the 2015 event are significantly higher than those in 1943, and ranged from 3 to 6 m along the coast from 29°S to 32°S, with localized peak values of 13 m at La Cebada (30.98°S, 71.65°W) and 10.8m at Totoral (30.37°S, 71.67°W) and a tide gauge peak recording of 4.5 m at Coquimbo to the north (119; 120). Far-field tsunami amplitudes reported in Japan for the 1943 event (10-30 cm, 91) are less than those reported in 2015 (11-80 cm). The macroseismic effects of the 1943 earthquake are broadly similar to the 2015 event, but extend further south. (8; 91). Aftershocks for the 1943 event, located by Kelleher (8) using S-P times from La Paz, indicate along-strike rupture zone dimension comparable to 2015 (106). Peak slip in 2015 (8-12 m) is greater than the slip accumulated during the interval 1943-2015 (5.3 m for 74 mm/yr convergence) although average slip is comparable. Overall, the 2015 event is not a simple repeat of the 1943 event and likely had much more slip at shallow depth (100). The 1880 rupture was similar in extent, but the 1730 rupture extended much further to the south, akin to the Ecuador-Colombia behavior. While there may be persistent asperities in the central and shallow megathrust, they may fail independently in some events and may participate in along-strike cascades in other events (106).

2010 Maule, Chile

The 27 February 2010 Maule (M_W 8.8) earthquake ruptured the plate boundary offshore of central Chile between 34°S and 38.5°S (Figure 1, 3f). The coseismic slip of this event has been determined by analysis of seismic, geodetic, and tsunami observations (121-134). Patchy coseismic slip is distributed over a region 460 km long and 100 km wide between the depths of 15 and 40 km. Two large-slip asperity regions are resolved along the megathrust, one extending from 34°S to 36°S (with up to 20 m slip) and the other from 37°S to 38°S (with up to 10 m slip). Finite fault inversions relying on only on-land static geodetic data tend to place slip on the central megathrust toward the coastline (124; 125; 131), but (132) and (134) find that the large-slip patches include slip of 5-8 m all the way to the trench based on joint inversions with accurately modeled tsunami observations. This is consistent with direct images of coseismic seafloor displacement at the trench from repeated seismic reflection surveys (135). Concentrations of outer trench-slope normal faulting occurred offshore from these shallow slip patches (132). Aftershocks concentrate along the down-dip megathrusts and around the large-slip zones (136).

Geodetic measurements had resolved accumulating slip deficit prior to the rupture along the entire rupture area, with moderate reduction near 35°S (95; 114; 123; 131; 137), but the patchy slip distribution only loosely conforms to the variable geodetic locking distribution (138). Afterslip extends along the length of the rupture primarily down-dip and between the two large coseismic slip patches (126; 131; 139-140). While the region was recognized as a seismic gap along the historic 1835 rupture zone and geophysical instrumentation was deployed in the region in advance of the earthquake, the co-seismic slip was moderate in the 1835 source area. Substantial slip overlapped the 1928 rupture zone and slip terminated adjacent to the 1985 rupture zone (141). The estimated slip deficit from 1835 to 2010 is ~12 m, somewhat above the average slip in the southern half of the rupture zone. Much less slip deficit could have accumulated after 1928, but that event could have ruptured the deeper megathrust, below the region of 20 m slip in 2010, with large ruptures in 1647, 1730 and 1751 possibly having ruptured the same region (Figure 2c). Conventional seismic gap ideas with strong segmentation do not characterize this

region well, but the Reid strain renewal concept in conjunction with a distribution of persistent asperities along the megathrust reconciles the historical behavior.

Southern Chile

Southern Chile (Figures 1, 2d), extending from 38°S near the Arauco Peninsula to 48°S near the intersection with the Chile Rise has hosted several great historic megathrust ruptures in 1575, 1737 and 1837 (52; 92; 142; 143), as well as the 1960 M_W 9.5 event (144-150). It appears that the 1737 and 1837 events had limited overlap (Figure 2d), and together spanned the 1575 and 1960 rupture extent (52). Paleotsunami evidence indicates ruptures preceding 1575 in 1337 ± 18(?) and 1154 ± 27 (94), with biostratigraphy giving compatible dates of 1270-1450 and 1070-1220 (151). A recurrence time of about 270 years appears to hold along this segment (142; 152). Dura et al. (152) also consider whether the Arauco Peninsula (37°-38°S) is a persistent barrier. The 2010 Maule event ruptured into, but not across this region, and the 1835, 1751, 1657 and 1570 events in Central Chile also did not cross it, nor did the 1960, 1737 and 1575 events to the south, so it appears to have been a persistent impediment to through-going rupture over the last 600 years.

Supplemental Refs

1. J.-M. Nocquet, J. C. Villegas-Lanza, M. Chlieh, P. A. Mothes, F. Rolandone, P. Jarrin et al., Motion of continental slivers and creeping subduction in the northern Andes. *Nat. Geosci*., 7, 287-291 (2014). https://doi.org/10.1038/NGEO2099.
2. M. Chlieh, P. A. Mothes, J.-M. Nocquet, P. Jarrin, P. Charvis, D. Cisneros, et al., Distribution of discrete seismic asperities and aseismic slip along the Ecuadorian megathrust. *Earth Planet. Sci. Lett.* 400, 292-301 (2014). https://doi.org/10.1016/j.epsl.2014.05.027.
3. A. Alvarado, L. Audin, J. M. Nocquet, E. Jaillard, P. Mothes, P. A. Jarrín, et al. Partitioning of oblique convergence in the Northern Andes subduction zone: Migration history and the present-day boundary of the North Andean Sliver in Ecuador. *Tectonics* 35, 1048-1065 (2016). https://doi.org/10.1002/2016TC004117.
4. P. Lonsdale, Ecuadorian subduction system. *Amer. Assoc. Pet. Geol. Bull*. 62, 2454-2477 (1978).
5. J.-Y. Collot, E. Sanclemente, J.-M. Nocquet, A. Lepretre, A. Riboderti, P. Jarrin, et al., Subducted oceanic relief locks the shallow megathrust in central Ecuador. *J. Geophys. Res*. 122, 3286-3305 (2017). https://doi.org/10.1002/2016JB013849
6. J.-M. Nocquet, P. Jarrin, M. Vallée, P. A. Mothes, R. Grandin, F. Rolandone et al., Supercycle at the Ecuadorian subduction zone revealed after the 2016 Pedernales earthquake. *Nat. Geosci*. 10, 145-149 (2017). https://doi.org/10.1038/NGEO2864.
7. P. A. Mothes, F. Rolandone, J.-M. Nocquet, P. A. Jarrín, A. P. Alvarado, M. C. Ruiz et al., Monitoring the earthquake cycle in the northern Andes from the Ecuadorian cGPS network. *Seism. Res. Lett.* 89, 534-541 (2018). https://doi.org/10.1785/0220170243.
8. J. A. Kelleher, Rupture zones of large South American earthquakes and some predictions. *J. Geophys. Res. 77*, 2087-2103 (1972).
9. K. Abe, Size of great earthquake of 1837-1979 inferred from tsunami data, *J. Geophys. Res*. 84, 1561-1568 (1979).
10. J. E. Ramirez, Earthquake history of Colombia, *Bull. Seism. Soc. Am*. 23, 13-22 (1933).
11. S. Migeon, C. Garibaldi, G. Ratzov, S. Schmidt, J.-Y. Collot, S. Zaragosi, L. Texier, Earthquake-triggered deposits in the subduction trench of the north Ecudaor/south Colombia margin and their implication for paleoseismology. *Mar. Geo.* 384, 47-62, (2017). https://doi.org/10.1016/j.margeo.2016.09.008.
12. H. Kanamori, K. C. McNally, Variable rupture mode of the subduction zone along the Ecuador-Colombia coast. *Bull. Seism. Soc. Am.* 72, 1241-1253 (1982).

13. C. Mendoza, J. W. Dewey, Seismicity associated with the great Colombia-Ecuador earthquakes of 1942, 1958, and 1979: Implications for barrier models of earthquake rupture. *Bull. Seism. Soc. Am*. 74, 577-593 (1984).
14. S. L. Beck, L.J. Ruff, The rupture process of the great 1979 Colombia earthquake: evidence for the asperity model. *J. Geophys. Res.* 89**,** 9281-9291 (1984).
15. L. Ye, H. Kanamori, J.-P. Avouac, L. Li, K. F. Cheung, T. Lay, The 16 April 2016, M_W 7.8 (M_S 7.5) Ecuador earthquake: A quasi-repeater of the 1942 M_S 7.5 earthquake and partial re-rupture of the 1906 M_S 8.6 Colombia-Ecuador earthquake. *Earth Planet. Sci. Lett*. 454, 248-258 (2016). https://doi.org/10.1016/j.epsl.2016.09.006.
16. P. He, E. A. Hetland, Q. Wang, K. Ding, Y. Wen, R. Zou, Coseismic slip in the 2016 M_W 7.8 Ecuador earthquake imaged from Sentinel-1A radar interferometry. *Seism. Res. Lett*. 88, 277-286 (2017). https://doi.org/10.1785/0220160151.
17. M. Heidarzadeh, S. Murotani, K. Satake, T. Takagawa, T. Saito, Fault size and depth extent of the Ecuador earthquake (M_W 7.8) of 16 April 2016 from teleseismic and tsunami data. *Geophys. Res. Lett*. 44, 2211-2219 (2017). https://doi.org/10.1002/2017GL072545.
18. M. Yoshimoto, M., H. Kumagai, W. Acero, G. Ponce, F. Vásconez, S. Arrais, et al., Depth-dependent rupture mode along the Ecuador-Colombia subduction zone. *Geophys. Res. Lett*. 44, 2203-2210 (2017). https://doi.org/10.1002/2016GL071929.
19. J. L. Swenson, S. L. Beck, Historical 1942 Ecuador and 1942 Peru subduction earthquakes, and earthquake cycles along Colombia-Ecuador and Peru subduction segments. *Pure Appl. Geophys.* 146, 67-101 (1996).
20. H. Perfettini, J.-P. Avouac, H. Tavera, A. Kositsky, J.-M. Nocquet, F. Bondoux, et al., Seismic and aseismic slip on the Central Peru megathrust. *Nature* 465, 78-81 (2010). https://doi.org/10.1038/nature09062.
21. J. C. Villegas-Lanza, M. Chlieh, O. Cavalié, H. Tavera, P. Baby, J. Chire-Chira, J.-M. Nocquet, Active tectonics of Peru: Heterogeneous interseismic coupling along the Nazca megathrust, rigid motion of the Peruvian Sliver, and Subandean shortening accommodation. J. *Geophys. Res.: Solid Earth* 121, 7371-7394 (2016). https://doi.org/10.1002/2016JB013080.
22. E. Silgado, Destructive earthquakes of South America 1530-1894, *Earthquake Mitigation Program in the Andean Region, Project SISRA,* vol. 10, 315 pp., Lima, Peru (1985).
23. L. Dorbath, A. Cisternas, C. Dorbath, Assessment of the size of large and great historical earthquakes in Peru. *Bull. Seism. Soc. Am.* 80, 551-576 (1990).
24. A. M. Pelayo, D. A. Wiens, The November 20, 1960 Peru tsunami earthquake – source mechanism of a slow event. *Geophys. Res. Lett.* 17, 661-664 (1990).

25. P. F. Ihmlé, J.-M. Gomez, P. Heinrich, S. Guibourg, The 1996 Peru tsunamigenic earthquake: Broadband source process. *Geophys. Res. Lett.* 25, 2691-2694 (1998).
26. S. L. Bilek, Seismicity along the South American subduction zone: Review of large earthquakes, tsunamis and subduction zone complexity. *Tectonophys.* 495, 2-14 (2010). https://doi.org/10.1016/j.tecto.2009.02.037.
27. Z. Wu, T. Lay, L. Ye, Shallow megathrust slip during large earthquakes that have high P coda levels. *J. Geophys. Res.: Solid Earth* 124, e2019JB018709. https://doi.org/10.1029/2019JB018709.
28. B. L. Askew, S. T. Algermissen (eds). *Catalog of Earthquakes for South America: Hypocenter and Intensity Data* (Ceresis publication, Volumes 4, 6, and 7a, b and c) (1985).
29. N. Wetzler, T. Lay, E. E. Brodsky, H. Kanamori, Rupture-depth-varying seismicity patterns for major and great (M_W ≥7.0) megathrust earthquakes. *Geophys. Res. Lett.* 44, 9663-9671 (2017). https://doi.org/10.1002/2017GL074573.
30. S. L. Beck, S.P. Nishenko, Variations in the mode of great earthquake rupture along the central Peru subduction zone. *Geophys. Res. Lett.* 17, 1969-1972 (1990).
31. J. W. Dewey, W. Spence, Seismic gaps and source zones of recent large earthquakes in coastal Peru. *Pure Appl. Geophys*., 117, 1148-1171 (1979).
32. S. L. Beck, L.J. Ruff, Great earthquakes and subduction along the Peru trench. *Phys. Earth Planet. Int.* 57, 199-224 (1989).
33. C. J. Langer, W. Spence, The 1974 Peru earthquake series. *Bull. Seism. Soc. Am.* 85, 665-687 (1995).
34. T. Lay, C. J. Ammon, A. R. Hutko, H. Kanamori, Effects of kinematic constraints on teleseismic finite-source rupture inversions: Great Peruvian earthquakes of 23 June 2001 and 15 August 2007. *Bull. Seism. Soc. Am.* 100, 969-994 (2010). https://doi.org/10.1785/0120090274.
35. A. Sladen, H. Tavera, M. Simons, J. P. Avouac, A. O. Konca, H. Perfettini, L. Audin, E. J. Fielding, F. Ortega, R. Cavagnoud, Source model of the 2007 M_w 8.0 Pisco, Peru earthquake: Implications for seismogenic behavior of subduction megathrusts. *J. Geophys. Res.* 115, B02405 (2010). https://doi.org/10.1029/2009JB006429.
36. M. Chlieh, H. Perfettini, H. Tavera, J.-P. Avouac, D. Remy, J.-M. Nocquet et al., Interseismic coupling and seismic potential along the Central Andes subduction zone. *J. Geophys. Res.* 116, B12405 (2011). https://doi.org/10.1029/2010JB008166.
37. M. E. Pritchard, E. O. Norabuena, C. Ji, R. Boroschek, D. Comte, M. Simons, T. H. Dixon, P. A. Rosen, Geodetic, teleseismic, and strong motion constraints on slip from recent southern Peru

subduction zone earthquakes. *J. Geophys. Res.* 112, B03307 (2007). https://doi.org/10.1029/2006JB004294.

38. M. Motagh, R. Wang, T. R. Walter, R. Bürgmann, E. Fielding, J. Anderssohn, J. Zschau, Coseismic slip model of the 2007 August Pisco earthquake (Peru) as constrained by wide swath radar observations. *Geophys. J. Int.* 174, 842-848 (2008).
39. M. E. Pritchard, E. J. Fielding, A study of the 2006 and 2007 earthquake sequence of Pisco, Peru, with InSAR and teleseismic data. *Geophys. Res. Lett.* 35, L09308 (2008). https://doi.org/10.1029/2008GL033374.
40. H. Tavera, I. Bernal, The Pisco (Peru) earthquake of 15 August 2007. *Seismol. Res. Lett.* 79, 510-515 (2008). https://doi.org/10.1785/gssrl.79.4.510.
41. J. D. Biggs, P. Robinson, T. H. Dixon, The 2007 Pisco, Peru, earthquake (M 8.0): seismology and geodesy. *Geophys. J. Int.* 176, 657-669 (2009). https://doi.org/10.1111/j.1365-246X.2008.03990.x.
42. O. Sufri, K. D. Koper, T. Lay, Along-dip seismic radiation segmentation during the 2007 M_W 8.0 Pisco, Peru earthquake. *Geophys. Res. Lett.* 39, L08311 (2012). https://doi.org/10.1029/2012GL051316.
43. M. Ioualalen, H. Perettini, S. Yauri Condo, C. Jimenez, H. Tavera, Tsunami modeling to validate slip models of the 2007 M_W 8.0 Pisco earthquake, Central Peru. *Pure Appl. Geophys.* 170, 433-451 (2013). https://doi.org/10.1007/s00024-012-0608-z.
44. D. Comte, M. Pardo, Reappraisal of great historical earthquakes in the Northern Chile and Southern Peru seismic gaps. *Nat. Haz.* 4, 23-44 (1991).
45. J. L. Swenson, S. L. Beck, Source characteristics of the 12 November 1996 M_W 7.7 Peru subduction zone earthquake. *Pure Appl. Geophys*. 154, 731-751 (1999).
46. M. K. Giovanni, S. L. Beck, L. Wagner (2002), The June 23, 2001 Peru earthquake and the southern Peru subduction zone. *Geophys. Res. Lett.* 29, 2018 (2002). https://doi.org/10.1029/2002GL015774.
47. S. L. Bilek, L. J. Ruff, Analysis of the 23 June 2001 $M_W = 8.4$ Peru underthrusting earthquake and its aftershocks. *Geophys. Res. Lett*. 29, 1960 (2002). https://doi.org/10.1029/2002GL015543.
48. D. P. Robinson, S. Das, A. B. Watts, Earthquake rupture stalled by a subducting fracture zone. *Science* 312, 1203-1205 (2006). https://doi.org/10.1126/science.1125771.
49. C. Jiménez, C. Carbonel, J. C. Villegas-Lanza, Seismic source of the earthquake of Camana Peru 2001 (M_W 8.2) from joint inversion of geodetic and tsunami data. *Pure Appl. Geophys*. 178, 4763-4775 (2021). https://doi.org/10.1007/s00024-020-02616-8.

50. H. Perfettini, J.-P. Avouac, J. Ruegg, Geodetic displacements and aftershocks following the 2001, M_W = 8.4 Peru earthquake: implications for the mechanics of the earthquake cycle along subduction zones. *J. Geophys. Res.* 110, B09404 (2005). https://doi.org/10.1029/2004JB003522.
51. J. P. Loveless, M. E. Pritchard, N. Kukowski, Testing mechanisms of subduction zone segmentation and seismogenesis with slip distributions from recent Andean earthquakes. *Tectonophys*. 495, 15-33 (2010). https://doi.org/10.1016/j.tecto.2009.05.008.
52. S. Ruiz, R. Madariaga, Historical and recent large megathrust earthquakes in Chile. *Tectonophys*. 733, 37-56 (2018). https://doi.org/10.1016/j.tecto.2018.01.015.
53. G. Vargas, L. Ortlieb, E Chapron, J. Valdes, C. Marquardt, Paleoseismic inerences from a high-resolution marine sedimentary record in northern Chile (23°S). *Tectonophys*. 399 381-398 (2005). https://doi.org/10.1016/j.tecto.2004.12.031.
54. M. Abad, T. Izquierdo, M. Cáceres, E. Bernández, J. Rodríguez-Vidal, Coastal boulder deposit as evidence of an ocean-wide prehistoric tsunami originated on the Atacama Desert coast (northern Chile). *Sedimentology* 67, 1505-1528 (2020).
55. I. Tsuji, Catalog of distant tsunamis researching Japan from Chile and Perú. *Rep. Tsunami Eng.* 30, 61-68 (2013).
56. D. Salazar, G. Easton, J. Goff, J. L. Guendon, J. González-Alfaro, P. Andrade, et al., Did a 3800-year-old M_W ~9.5 earthquake trigger major social disruption in the Atacama desert? *Sci. Adv.* 8, eabm2996 (2022). https://doi.org/10.1126/sciadv.abm2996.
57. C. An, I. Sepúlveda, P. L.-F. Liu, Tsunami source and its validation of the 2014 Iquique, Chile, earthquake. *Geophys. Res. Lett.*, 41 3988-3994 (2014). https://doi.org/10.1002/2014GL060567.
58. G. P. Hayes, M. W. Herman, W. D. Barnhart, K. P. Furlong, S. Riquelme, H. M. Benz, et al., Continuing megathrust earthquake potential in Chile after the 2014 Iquique earthquake. *Nature* 512, 295-298 (2014). https://doi.org/10.1038/nature13677.
59. T. Lay, H. Yue, E. E. Brodsky, C. An, The 1 April 2014 Iquique, Chile M_W 8.1 earthquake rupture sequence. *Geophys. Res. Lett.* 41, 3818-3825 (2014). https://doi.org/10.1002/2014GL060238.
60. Y. Bai, K. F. Cheung, Y. Yamazaki, T. Lay, L. Ye, Tsunami surges around the Hawaiian Islands from the 1 April 2014 North Chile M_W 8.1 earthquake. *Geophys. Res. Lett.* 41, 8512-8521 (2014). https://doi.org/10.1002/2014GL061686.
61. Y. Yagi, R. Okuwaki, B. Enescu, S. Hirano, Y. Yamagami, S. Endo, T. Komoro, Rupture process of the 2014 Iquique Chile Earthquake in relation with the foreshock activity. *Geophys. Res. Lett.* 41, 4201-4206 (2014). https://doi.org/10.1002/2014GL060274.

62. A. R. Gusman, S. Murotani, K. Satake, M. Heidarzadeh, E. Gunawan, S. Watada, B. Schurr, Fault slip distribution of the 2014 Iquique, Chile earthquake estimated from ocean-wide tsunami waveforms and GPS data. *Geophys. Res. Lett.* 42, 1053-1060 (2015). https://doi.org/10.1002/2014GL062604.
63. Z. Duputel, J. Jiang, R. Jolivet, M. Simons, L. Rivera, J.-P. Ampuero, et al., The Iquique earthquake sequence of April 2014: Bayesian modeling accounting for prediction uncertainty. *Geophys. Res. Lett.* 42, 7949-7957 (2015). https://doi.org/10.1002/2015GL065402.
64. C. Liu, Y. Zheng, R. Wang, X. Xiong, Kinematic rupture process of the 2014 Chile M_W 8.1 earthquake constrained by strong-motion, GPS static offsets and teleseismic data. *Geophys. J. Int.* 202, 1137-1145 (2015). https://doi.org/10.1093/gji/ggv214.
65. E. E. Brodsky, T. Lay, Recognizing foreshocks from the 1 April 2014 Chile earthquake, *Science* 344, 700-702 (2014). https://doi.org/10.1126/science.1255202.
66. A. Kato, S. Nakagawa, Multiple slow-slip events during a foreshock sequence of the 2014 Iquique, Chile M_W 8.1 earthquake. *Geophys. Res. Lett.* 41, 5420-5427 (2014). https://doi.org/10.1002/2014GL061138.
67. S. Ruiz, M. Métois, A. Fuenzalida, J. Ruiz, F. Leyton, R. Grandin, C. Vigny, R. Madariaga, J. Campos, Intense foreshocks and a slow slip event preceded the 2014 Iquique M_W 8.1 earthquake. *Science* 345, 1165-1169 (2014). https://doi.org/10.1126/science.1256074.
68. B. Schurr, G. Asch, S. Hainzl, J. Bedford, A. Hoechner, M. Palo, et al., Gradual unlocking of plate boundary controlled initiation of the 2014 Iquique earthquake. *Nature* 512, 299-302 (2014). https://doi.org/10.1038/nature13681.
69. L. Meng, H. Huang, R. Bürgmann, J. P. Ampuero, A. Strader, Dual megathrust slip behaviors of the 2014 Iquique earthquake sequence. *Earth Plant. Sci. Lett.* 411, 177-187 (2015). https://doi.org/10.1016/j.epsl.2014.11.041.
70. S. Cesca, F. Grigoli, S. Heimann, T. Dahmn, M. Kriegerowski, M. Sobiesisak, et al., The M_W 8.1 2014 Iquique, Chile, seismic sequence: a tale of foreshocks and aftershocks. *Geophys. J. Int.* 204, 1766-1780 (2016). https://doi.org/10.1093/gji/ggv544.
71. A. Socquet, J. P. Valdes, J. Jara, F. Cotton, A. Walpersdorf, N. Cotte, et al., An 8 months slow slip event triggers progressive nucleation of the 2014 Chile megathrust. *Geophys. Res. Lett.* 44, 4046-4053 (2017). https://doi.org/10.1002/2017GL073023.
72. F. Hoffmann, S. Metzger, M. Moreno, Z. Deng, C. Sippl, F. Ortega-Culaciati, O. Oncken, Characterizing afterslip and ground displacement rate increase following the 2014 Iquique-Pisagua M_W 8.1 earthquake, Northern Chile. *J. Geophys. Res.: Solid Earth* 123, 4171-4192 (2018). https://doi.org/10.1002/2017JB014970.

73. M. Chlieh, J. B. de Chabalier, J. C. Ruegg, R. Armijo, R. Dmowska, J. Campos, K. L. Feigl, Crustal deformation and fault slip during the seismic cycle in the North Chile subduction zone, from GPS and InSAR observations. *Geophys. J. Int.* 158, 695-711 (2004). https://doi.org/10.1111/j.1365-246X.2004.02326.x.
74. M. Métois, A. Socquet, C. Vigny, D. Carrizo, S. Peyrat, A. Delorme, et al., Revisiting the North Chile seismic gap segmentation using GPS-derived interseismic coupling. *Geophys. J. Int.* 194, 1283-1294 (2013). https://doi.org/10.1093/gji/ggt183.
75. E. Kausel, Los terremotos de Agosto de 1868 y Mayo de 1877 que afectaron el sur del Perú y norte de Chile. *Boletín de la Academia Chilena de Ciencias* 3, 8-13 (1986).
76. C. Vigny, E. Klein, The 1877 megathrust earthquake of North Chile two times smaller than thought? A review of ancient articles. *J. S. Amer. Earth Sci.* 117, 103878 (2022). https://doi.org/10.1016/j.jsames.2022.103878.
77. Delouis, B., M. Pardo, D. Legrand, T. Monfret, The M_W 7.7 Tocopilla earthquake of 14 November 2007 at the southern edge of the northern Chile seismic gap: Rupture in the deep part of the coupled plate interface. *Bull. Seism. Soc. Am.* 99, 87-94 (2009). https://doi.org/10.1785/0120080192.
78. M. Béjar-Pizarro, Asperities and barriers on the seismogenic zone in North Chile: state-of-the-art after the 2007 M_W 7.7 Tocopilla earthquake inferred by GPS and InSAR data. *Geohys. J. Int.* 183, 390-406 (2010). https://doi.org/10.1111/j.1365-246X.2010.04748.x
79. S. Peyrat, R. Madariaga, E. Buforn, J. Campos, G. Asch, J. P. Vilotte, Kinematic rupture process of the 2007 Tocopilla earthquake and its main aftershocks from teleseismic and strong motion data. *Geophys. J. Int,* 182, 1411-1430 (2010). https://doi.org/10.1111/j.1365-246X.2010.04685.x.
80. B. Schurr, G. Asch, M. Rosenau, R. Wang, O. Oncken, S. Barrientos, P. Salazar, J.-P. Vilotte, The 2007 *M*7.7 Tocopilla northern Chile earthquake sequence: Implications for along-strike and downdip rupture segmentation and megathrust frictional behavior. *J. Geophys. Res.* 117*,* B05305 (2012). https://doi.org/10.1029/2011JB009030.
81. J. C. Ruegg, J. Campos, R. Armijo, S. Barrientos, P. Briole, R. Thiele, et al., The M_W=8.1 Antofagasta (North Chile) earthquake of July 30, 1995: First results from teleseismic and geodetic data. *Geophys. Res. Lett.* 23, 917-920 (1996).
82. Delouis, B., T. Monfret, L. Dorbath, M. Pardo, L. Rivera, D. Comte, et al., The M_W = 8.0 Antofagasta (Northern Chile) earthquake of 30 July 1995: A precursor to the end of the larger 1877 gap. *Bull. Seism. Soc. Am.* 87, 427-445 (1997).

83. P. F. Ihmlé, J.-C. Ruegg, Source tomography by simulated annealing using broad-band surface waves and geodetic data: application to the M_W=8.1 Chile 1995 event. *Geophys. J. Int.* 131, 146-158 (1997).
84. S. Guibourg, P. Heinrich, R. Roche, Numerical modeling of the 1995 Chilean tsunami. Impact on French Polynesia. *Geophys. Res. Lett*. 24, 775-778 (1997).
85. D. L. Carlo, T. Lay, C. J. Ammon, J. Zhang, Rupture process of the 1995 Antofagasta subduction earthquake (M_W = 8.1). *Pure Appl. Geophys*. 154 677-709 (1999).
86. J. Klotz, D. Angermann, G. W. Michel, R. Porth, C. Reigber, J. Reinking, et al., GPS-derived deformation of the central Andes including the 1995 Antofagasta M_W = 8.0 earthquake. Pure Appl. Geophys. 154 709-730 (1999).
87. M. E. Pritchard, M. Simons, P. A. Rosen, S. Hensley, F. H. Webb, Co-seismic slip rom the 1995 July 30 M_W=8.1 Antofagasta, Chile, earthquake as constrained by InSAR and GPS observations. *Geophys. J. Int.* 150, 362-376 (2002).
88. M. E. Pritchard, C. Ji, M. Simons, Distribution of slip from 11 M_W > 6 earthquake in the northern Chile subduction zone, *J. Geophys. Res*. 111, B10302 (2006). https://doi.org/10.1029/2005JB004013.
89. M.E. Pritchard, M. Simons, An aseismic slip pulse in northern Chile and along-strike variations in seismogenic behavior. *J. Geophys. Res*. 111, B08405 (2006). https://doi.org/10.1029/2006JB004258.
90. P. F. Ihmlé, R. Madariaga, Monochromatic body waves excited by great subduction zone earthquakes. *Geophys. Res. Lett*. 23, 2999-3002 (1996).
91. S. Beck, S. Barrientos, E. Kausel, M. Reyes, Source characteristics of historic earthquakes along the central Chile subduction zone. *J. South Am. Earth Sci.* 11, 115–129 (1998).
92. C. Lomnitz, C., Major earthquakes of Chile: A historical survey, 1535 – 1960. *Seism. Res. Lett.* 75, 368-378 (2004). https://doi.org/10.1785/gssrl.75.3.368.
93. H. Kanamori, L. Rivera, L. Ye, T. Lay, S. Murotani, K. Tsumura, New constraints on the 1922 Atacama, Chile, earthquake from historical seismograms. *Geophys. J. Int*. 219, 645-661 (2019). https://doi.org/10.1093/gji/ggz302.
94. G. Easton, J. González-Alfaro, A. Villalobos, G. Álvarez, D. Melgar, S. Ruiz, B. Sepúlveda, M. Escobar, T. León, J. Carlos Báez, et al., Complex rupture of the 2015 M_W 8.3 Illapel earthquake and prehistoric events in the Central Chile tsunami gap. *Seis. Res. Lett*. 93, 1479–1496 (2022). https://doi.org/10.1785/0220210283.

95. M. Métois, A. Socquet, C. Vigny, Interseismic coupling, segmentation and mechanical behavior of the central Chile subduction zone. *J. Geophys. Res.* 117, B03406 (2012). https://doi.org/10.1029/2011JB008736.
96. M. Métois, C. Vigny, A. Socquet, A. Delorme, S. Morvan, I. Ortega, C. M. Valderas-Bermejo, GPS-derived interseismic coupling on the subduction and seismic hazards in the Atacama region, Chile, *Geophys. J. Inter.*, 196, 644-655 (2014). https://doi.org/10.1093/gji/ggt418.
97. M. W. Herman, R. Govers, Locating fully locked asperities along the South America subduction megathrust: A new physical inter-seismic inversion approach in a Bayesian framework. *Geochem. Geophys., Geosys*. 21, e2020GC009063 (2020).
98. C. Lomnitz, Major earthquakes and tsunamis in Chile during the period 1535 to 1953. *Geol. Rundsch*. 59, 938-960 (1970).
99. S. P. Nishenko, Seismic potential for large and great interplate earthquakes along the Chilean and southern Peruvian margins of South America: A quantitative reappraisal. *J. Geophys. Res.* 90, 3589–3615 (1985). https://doi.org/10.1029/JB090iB05p03589.
100. L. Ye, T. Lay, H. Kanamori, K.D. Koper, Rapidly estimated seismic source parameters for the 16 September 2015 Illapel Chile M_W 8.3 earthquake. *Pure Appl. Geophys.* 173, 321-332 (2016). https://doi.org/10.1007/s00024-015-1202-y.
101. M. Heidarzadeh, S. Murotani, K. Satake, T. Ishibe, A. R. Gusman, Source model of the 16 September 2015 Illapel, Chile M_W 8.4 earthquake based on teleseismic and tsunami data. *Geophys. Res. Lett*. 43, 643-650 (2016). https://doi.org/10.1002/2015GL067297.
102. L. Li, T. Lay, K. F. Cheung, L. Ye, Joint modeling of teleseismic and tsunami wave observations to constrain the 16 September 2015 Illapel, Chile, M_W 8.3 earthquake rupture process. *Geophys. Res. Lett.* 43, 4303-4312 (2016). https://doi.org/10.1002/2016GL068674.
103. D. Melgar, W. Fan, S. Riquelme, J. Geng, C. Liang, M. Fuentes, G. Vargas, R. M. Allen, P. M. Shearer, E. J. Fielding, Slip segmentation and slow rupture to the trench during the 2015, M_W8.3 Illapel, Chile earthquake. *Geophys. Res. Lett.* 43, 961–966 (2016). https://doi.org/10.1002/2015GL067369.
104. R. Okuwaki, Y. Yagi, R. Aránguiz, J. González, G. González, Rupture process during the 2015 Illapel, Chile Earthquake: Zigzag-along-dip rupture episodes. *Pure. Appl. Geophys*. 173, 1011-1020 (2016). https://doi.org/10.1007/s00024-016-1271-6.
105. S. Ruiz, E. Klein, F. del Campo, E. Rivera, P. Poli, M. Metois, V. Christophe, J.C. Baez, G. Vargas, F. Leyton, R. Madariaga, L. Fleitout, The seismic sequence of the 16 September 2015 M_W 8.3 Illapel, Chile earthquake. *Seism. Res. Lett.* 87, 789-799 (2016). https://doi.org/10.1785/0220150281.

106. F. Tilmann, Y. Zhang, M. Moreno, J. Saul, F. Eckelmann, M. Palo et al., The 2015 Illapel earthquake, central Chile: A type case for a characteristic earthquake? *Geophys. Res. Lett.* 43, 574–583 (2016). https://doi.org/10.1002/2015GL066963.

107. J. Yin, H. Yang, H. Yao, H. Weng, Coseismic radiation and stress drop during the 2015 M_W 8.3 Illapel, Chile megathrust earthquake. *Geophys. Res. Lett.* 43, 1520-1528 (2016). https://doi.org/10.1002/2015GL067381.

108. C. An, H. Yue, J. Sun, L. Meng, J. C. Báez, The 2015 M_W 8.3 Illapel, Chile, earthquake: Direction-reversed along-dip rupture with localized water reverberation. *Bull. Seism. Soc. Am.*, 107, 2416-2426 (2017). https://doi.org/10.1785/0120160393.

109. M. W. Herman, J. L. Nealy, W. L. Yeck, W. D. Barnhart, G. P. Hayes, K. P. Furlong, H. M. Benz, Integrated geophysical characteristics of the 2015 Illapel, Chile, earthquake. *J. Geophys. Res.: Solid Earth* 122, 4691-4711 (2017). https://doi.org/10.1002/2016JB013617.

110. E. Klein, C. Vigny, L. Fleitout, R. Grandin, R. Jolivet, E. Rivera, M. Métois, A comprehensive analysis of the Illapel 2015 M_W 8.3 earthquake from GPS and InSAR data. *Earth Planet. Sci. Lett.* 469, 123-134 (2017). https://doi.org/10.1016/j.epsl.2017.04.010.

111. K. Satake, M. Heidarzadeh, A review of source models of the 2015 Illapel, Chile earthquake and insights from tsunami data. *Pure Appl. Geophys*. 174, 1-9 (2017). https://doi.org/10.1007/s00024-016-1450-5.

112. A. Williamson, A. Newman, and P. Cummins, Reconstruction of coseismic slip from the 2015 Illapel earthquake using combined geodetic and tsunami waveform data/ *J. Geophys. Res. Solid Earth* 122, 2119-2130 (2017). https://doi.org/10.1002/2016JB013883.

113. C. Liu, C. An, B. Shan, X. Xiong, X. Chen, Insights into the kinematic rupture of the 2015 M_W 8.3 Illapel, Chile, earthquake from joint analysis of geodetic, seismological, tsunami, and superconductive gravimeter observations. *J. Geophys. Res.: Solid Earth* 123, 9778-7999 (2018). https://doi.org/10.1029/2018JB016065.

114. C. Vigny, A. Rudloff, J.-C. Ruegg, R. Madariaga, J. Campos, M. Alvarez, Upper plate deformation measured by GPS in the Coquimbo Gap, Chile. *Phys. Earth Planet. Inter.* 175, 86–95 (2009). https://doi.org/10.1016/j.pepi.2008.02.013.

115. M. Métois, C. Vigny, A. Socquet, Interseismic coupling, megathrust earthquakes and seismic swarms along the Chilean subduction zone (38°-18°S). *Pure Appl. Geophys.* 1783, 1431-1449 (2016).

116. M. N. Shrivastava, G. González, M. Moreno, M. Chlieh, P. Salazar, C. D. Reddy, et al., Coseismic slip and afterslip of the 2015 M_W 8.3 Illapel (Chile) earthquake determined from

continuous GPS data. *Geophys. Res. Lett.* 43, 10710-10719 (2016). https://doi.org/10.1002/2016GL070684.

117. W. Feng, S. Samsonov, Y. Tian, Q. Qiu, P. Li, Y. Zhang, Z. Deng, K. Omari, Surface deformation associated with the 2015 M_W 8.3 Illapel earthquake revealed by satellite-based geodetic obseervations and its implications for the seismic cycle. *Earth Planet. Sci. Lett.* 460, 222-233 (2017). https://doi.org/10.1016/j.epsl.2016.11.018.

118. H. Huang, W. Xu, L. Meng, R. Burgmann, J. C. Baez, Early aftershocks and afterslip surrounding the 2015 M_W 8.4 Illapel rupture. *Earth Planet Sci. Lett.* 457, 282-291 (2017). https://doi.org/10.1016/j.epsl.2016.09.055.

119. R. Aránguiz, G. González, J. González, P. A. Catalán, R. Cienfuegos, Y. Yagi, et al., The 16 September 2015 Chile tsunami from the post-tsunami survey and numerical modeling perspectives, *Pure Appl. Geophys*. 333-348 (2016). https://doi.org/10.1007/s00024-015-1225-4.

120. M. Contreras-López, P. Winckler, I. Sepúlveda, A. Anduar-Álvarez, F. Cortés-Molina, C. J. Gueerrero, et al., Field survey of the 2015 Chile tsunami with emphasis on coastal wetland and conservation areas. *Pure Appl. Geophys*. 173, 349-367 (2016). https://doi.org/10.1007/s00024-015-1235-2.

121. B. Delouis, J.-M. Nocquet, M. Vallée, Slip distribution of the February 27, 2010 $M_W = 8.8$ Maule earthquake, central Chile, from static and high-rate GPS, InSAR and broadband teleseismic data. *Geophys. Res. Lett*. 37, L17305 (2010). https://doi.org/10.1029/2010GL043899.

122. T. Lay, C. J. Ammon, H. Kanamori, K. D. Koper, O. Sufri, A. R. Hutko, Teleseismic inversion for rupture process of the 27 February 2010 Chile (M_W 8.8) earthquake. *Geophys. Res. Lett*. 37, L11301 (2010). https://doi.org/10.1029/2010GL043379.

123. M. Moreno, M. Rosenau, O. Oncken, 2010 Maule earthquake slip correlates with pre-seismic locking of Andean subduction zone. *Nature* 467, 198–202 (2010). https://doi.org/10.1038/nature09349.

124. X. Tong, D. Sandwell, K. Luttrell, B. Brooks, M. Bevis, M. Shimada, et al., The 2010 Maule, Chile earthquake: Downdip rupture limit revealed by space geodesy. *Geophys. Res. Lett.* 37, L24311 (2010). https://doi.org/10.1029/2010GL045805.

125. F. Pollitz, B. Brooks, X. Tong, M. G. Bevis, J. H. Foster, R. Bürgmann, et al., Coseismic slip distribution of the February 27, 2010 M_W 8.8 Maule, Chile earthquake. *Geophys. Res. Lett*. 38, L09309 (2011). https://doi.org/10.1029/2011GL047065.

126. C. Vigny, A. Socquet, S. Peyrat, J.-C. Ruegg, M. Métois, R. Madariaga, et al., The 2010 M_W 8.8 Maule mega-thrust earthquake of central Chile, monitored by GPS. *Science 332*, 1417-1421 (2011). https://doi.org/10.1126/science.1204132.

127. Y. Fujii, K. Satake, Slip distribution and seismic moment of the 2010 and 1960 Chilean earthquakes inferred from tsunami waveforms and coastal geodetic data. *Pure Appl. Geophys*. 170, 1493-1509 (2013). https://doi.org/10.1007/s00024-012-0524-2.

128. K. D. Koper, A. R. Hutko, T. Lay, O. Sufri, Imaging short-period seismic radiation from the 27 February 2010 Chile (M_W 8.8) earthquake by back-projection of P, PP, and PKIKP waves. *J. Geophys. Res: Solid Earth* 117, B02308 (2012). https://doi.org/10.1029/2011JB008576.

129. M. Moreno, D. Melnick, M. Rosenau, J. Baez, J. Klotz, O. Oncken, et al., Toward understanding tectonic control on the M_W 8.8 2010 Maule Chile earthquake. *Earth Planet. Sci. Lett.* 321-322, 152-165 (2012). https://doi.org/10.1016/j.epsl.2012.01.006.

130. G. P. Hayes, E. Bergman, K. L Johnson, H. M. Benz, L. Brown, A. S. Meltzer, Seismotectonic framework of the 2010 February 27 M_W 8.8 Maule, Chile earthquake sequence. *Geophys. J. Int*. 195, 1034-1051 (2013). https://doi.org/10.1093/gji/ggt238.

131. Y. N. Lin, A. Sladen, F. Ortega-Culaciati, M. Simons, J.-P. Avouac, E. J. Fielding, et al., Coseismic and postseismic slip associated with the 2010 Maule earthquake, Chile: characterizing the Arauco Peninsula barrier effect. *J. Geophys. Res.: Solid Earth* 118, 3142-3159 (2013). https://doi.org/10.1002/jgrb.50207.

132. H, Yue, T. Lay, L. Rivera, C. An, C. Vigny, X. Tong, J. C. Báez Soto, Localized fault slip to the trench in the 2010 Maule, Chile M_w 8.8 earthquake from joint inversion of high-rate GPS, teleseismic body waves, InSAR, campaign GPS, and tsunami observations. *J. Geophys. Res*. 119, 7786-7804 (2014). https://doi.org/10.1002/2014JB011340.

133. M. Yoshimoto, S. Watada, Y. Fujii, K. Satake, Source estimate and tsunami forecast from far-field deep-ocean tsunami waveforms – The 27 February 2010 M_W 8.8 Maule earthquake. *Geophys. Res. Lett. 43*, 659-665 (2016). https://doi.org/10.1002/2015GL067181.

134. F. Romano, S. Lorito, T. Lay, A. Piatanesi, M. Volpe, S. Murphy, R. Tonini, Benchmarking the optimal time alignment of tsunami waveforms in nonlinear joint inversions for the M_W 8.8 2010 Maule (Chile) earthquake. *Frontiers in Earth Science* 8, 585429 (2020). https://doi.org/10.3389/feart.2020.585429.

135. A. Maksymowicz, C. D. Chadwell, J. Ruiz, A. M. Tréhu, E. Contreras-Reyes, W. Weinrebe, et al., Coseismic seafloor deformation in the trench region during the M_W 8.8 Maule megthrust earthquake. *Sci. Rep*., 7, 45918 (2017). https://doi.org/10.1038/srep45918.

136. A. Rietbrock, I. Ryder, G. Hayes, C. Haberland, D. Comte, S. Roecker, H. Lyon-Caen, Aftershock seismicity of the 2010 Maule M_W = 8.8, Chile, earthquake: Correlation between co-seismic slip models and aftershock distribution? *Geophys. Res. Lett. 39*, L08310 (2012). https://doi.org/10.1029/2012GL051308.

137. J. C. Ruegg, J. C., A. Rudloff, C. Vigny, R. Madariaga, J. B. de Chabalier, J. Campos, E. Kausel, S. Barrientos, D. Dimitrov, Interseismic strain accumulation measured by GPS in the seismic gap between Constitución and Concepción in Chile. *Phys. Earth Planet Inter.* 175, 78-85 (2009). https://doi.org/10.1016/j.pepi.2008.02.015.

138. S. Lorito, F. Romano, S. Atzori, X. Tong, A. Avallone, J. McCloskey, et al., Limited overlap between the seismic gap and coseismic slip of the great 2010 Chile earthquake. *Nature Geoscience* 4**,** 173-177 (2011). https://doi.org/10.1038/ngeo1073.

139. J. Bedford, M. Moreno, J. C. Baez, D. Lange, F. Tilmann, M. Rosenau, C. Vigny, A high-resolution, time-variable afterslip model for the 2010 Maule M_W = 8.8, Chile megathrust earthquake. *Earth Planet. Sci. Lett. 383*, 26–36 (2013). https://doi.org/10.1016/j.epsl.2013.09.020.

140. E. Klein, L. Fleitout, C. Vigny, J. D. Garaud, Afterslip and viscoelastic relaxation model inferred rom the large-scale post-seismic deformation following the 2010 M_W 8.8 Maule earthquake (Chile). *Geophys. J. Int.* 205, 1455-1472 (2016). https://doi.org/10.1093/gji/ggw086.

141. D. Comte, A. Eisenberg, E. Lorac, M. Pardo, L. Ponce, R. Saragoni, S. K. Singh, G. Suarez, The 1985 central Chile earthquake: A repeat of previous great earthquakes in the region? *Science* 233, 449–453 (1986).

142. M. Cisternas, B. F. Atwater, F. Rorrejón, Y. Sawai, G. Machuca, M. Lagos, A. Eipert, C. Youlton, I. Salgado, T. Kamataki, et al., Predecessors of the giant 1960 Chile earthquake. *Nature* 437, 404-407 (2005).

143. M. Cisternas, M. Carvajal, R. Wesson, L. L. Ely, N. Gorigoitia, Exploring the historical earthquakes preceding the giant 1960 Chile earthquake in a time-dependent seismogenic zone. *Bull. Seism. Soc. Am*. 107, 2664-2675 (2017). https://doi.org/10.1785/0120170103.

144. G. Plafker, J. Savage, Mechanism of the Chilean earthquake of May 21 and 22, 1960. *Geol. Soc. Am. Bull.* 81, 1001-1030 (1970).

145. G. Plafker, Alaskan earthquake of 1964 and Chilean earthquake of 1960: Implications for arc tectonics. *J. Geophys. Res.* 77, 901-925 (1972).

146. H. Kanamori, J. Cipar, Focal process of the great Chilean earthquake May 22, 1960. *Phys. Earth Planet Inter.* 9, 128-136 (1974).

147. I. L. Cifuentes, P. G. Silver, Low-frequency source characteristics of the great 1960 Chilean earthquake. *J. Geophys. Res*. 94, 643-663 (1989).

148. S. Barrientos, S. Ward, The 1960 Chile earthquake: Inversion for slip distribution from surface deformation. *Geophys. J. Int*. 103, 589-598 (1990).

149. M. S. Moreno, J. Bolte, J. Klotz, D. Melnick, Impact of megathrust geometry on inversion of coseismic slip from geodetic data: application to the 1960 Chile earthquake. *Geophys. Res. Lett.* 36, L16310 (2009). https://doi.org/10.1029/2009GL039276.

150. H. Kanamori, L. Rivera, S. Lambotte, Evidence for a large strike-slip component during the 1960 Chilean earthquake. Geophys. J. Int., 218, 1-32 (2019). https://doi.org/10.1093/gji/ggz113.

151. E. Garrett, I. Shennan, S. A. Woodroe, M. Cisternas, E. P. Hocking, P. Gulliver, Reconstructing paleoseismic deformation, 2: 1000 years of great earthquakes at Chucalén, south central Chile. *Quat. Sci. Rev*. 113, 112-122 (2015). https://doi.org/10.1016/j.quascirev.2014.10.010.

152. T. Dura, B. P. Horton, M. Cisternas, L. L. Ely, I Hong, A. R. Nelson, et al., Subduction zone slip variability during the last millennium, south –central Chile. *Quat. Sci. Rev* 175, 112-137 (2017). https://doi.org/10.1016/j.quascirev.2017.08.023. .
